# Mixed effects modeling of radiotherapy in combination with immune checkpoint blockade or inhibitors of the DNA damage response pathway

**DOI:** 10.1002/psp4.13026

**Published:** 2023-09-18

**Authors:** David Hodson, Hitesh Mistry, Sofia Guzzetti, Michael Davies, Anna Staniszewska, Paul Farrington, Elaine Cadogan, James Yates, Leon Aarons, Kayode Ogungbenro

**Affiliations:** ^1^ Division of Pharmacy and Optometry, Faculty of Biology, Medicine and Health The University of Manchester Manchester UK; ^2^ DMPK, Research and Early Development, Oncology R&D AstraZeneca Cambridge UK; ^3^ DMPK, Research and Early Development, Neuroscience R&D AstraZeneca Cambridge UK; ^4^ Bioscience, Research and Early Development, Oncology R&D AstraZeneca Cambridge UK; ^5^ GlaxoSmithKline – Stevenage Stevenage UK

## Abstract

Dosage optimization to maximize efficacy and minimize toxicity is a potential issue when administering radiotherapy (RT) in combination with immune checkpoint blockade (ICB) or inhibitors of the DNA Damage Response Pathway (DDRi) in the clinic. Preclinical models and mathematical modeling can help identify ideal dosage schedules to observe beneficial effects of a tri‐therapy. The aim of this study is to describe a mathematical model to capture the impact of RT in combination with inhibitors of the DNA Damage Response Pathway or blockade of the immune checkpoint protein – programmed death ligand 1 (PD‐L1). This model describes how RT mediated activation of antigen presenting cells can induce an increase in cytolytic T cells capable of targeting tumor cells, and how combination drugs can potentiate the immune response by inhibiting the rate of T cell exhaustion. The model was fitted using preclinical data, where MC38 tumors were treated in vivo with RT alone or in combination with anti‐PD‐L1 as well as with either olaparib or the ataxia telangiectasia mutated (ATM) inhibitor—AZD0156. The model successfully described the observed data and goodness‐of‐fit, using visual predictive checks also confirmed a successful internal model validation for each treatment modality. The results demonstrated that the anti‐PD‐L1 effect in combination with RT was maximal in vivo and any additional benefit of DDRi at the given dosage and schedule used was undetectable. Model fit results indicated AZD0156 to be a more potent DDRi than olaparib. Simulations of alternative doses indicated that reducing efficacy of anti‐PD‐L1 by 68% would potentially provide evidence for a benefit of ATM inhibition in combination with ICB and increase the relative efficacy of tri‐therapy.

**Study Highlights**

**WHAT IS THE CURRENT KNOWLEDGE OF THIS TOPIC?**

Clinical trials incorporating radiotherapy (RT) in combination with both immune checkpoint blockade (ICB) and DNA damage response pathway inhibitor (DDRi) are ongoing. Currently, there are very few mathematical models which may provide information on optimizing the dosage and schedule for these drugs.

**WHAT QUESTION DID THIS STUDY ADDRESS?**

This study uses nonlinear mixed effects modeling to capture the differential effects of RT in combination with ICB and/or DDRi on a population of mice challenged with the syngeneic tumor model – MC38.

**WHAT DOES THIS STUDY ADD TO OUR KNOWLEDGE?**

This study provides a new mathematical framework for assessing the impact of RT in combination with additional therapies on tumor growth trajectories. The model also allows for simulation of alternative potencies that may show improved relative responses of tri‐therapy compared with bi‐therapies.

**HOW MIGHT THIS CHANGE DRUG DISCOVERY, DEVELOPMENT, AND/OR THERAPEUTICS?**

This model provides potential scope for further assessment of potential dosage regimens that could improve the relative efficacy of tri‐therapy. This could be beneficial for optimizing dosing strategies in preclinical experiments.

## INTRODUCTION

The first programmed death ligand 1 (PD‐L1) inhibitor, atezolizumab was approved by the US Food and Drug Administration in 2016^1^ and, since then, other anti‐PD‐L1 agents, such as durvalumab^2^ and avelumab,^3^ have shown to have significant clinical benefits in a wide variety of cancers, such as urothelial carcinoma,^1^, ^2^, ^3^ non‐small cell lung cancer (NSCLC),^4^ and colorectal cancer.^5^ Recent studies, however, have indicated that in patients with NSCLC, anti‐PD‐L1 based therapies are mostly effective in tumors with high tumor associated expression of PD‐L1, where median overall survival is shown to increase significantly compared with standard of care platinum‐based chemotherapy. However, other tumors, such as hepatocellular carcinoma, do not share this link between PD‐L1 expression and survival after treatment with anti‐PD‐L1.^6^ Furthermore, improvements in progression‐free survival are not always significant, this is partially due to immunoresponsive tumors having a strong propensity to relapse when immunotherapy is administered.^7^ Tumors can attain acquired immunity to anti‐PD‐L1 via various mechanisms, such as the acquired upregulation of different exhaustion receptors capable of further reducing the cytolytic activity of effector T cells.^8^

Although PD‐L1 targeted immune checkpoint blockade (ICB) has shown some capability in prolonging survival for patients with metastatic cancer when administered as a monotherapy or in combination with chemotherapy,^9^ another potential combination partner to anti‐PD‐L1 may include radiotherapy (RT), which may provide additional curative benefits in the clinic. RT is known to induce expression of damage‐associated molecular patterns which are known to play significant roles in the activation of antigen presenting cells (APCs).^10^ Dendritic cells then present tumor‐associated antigen to CD8^+^ T cells, where the CD8^+^ T cell differentiates into a cytotoxic T cell, capable of inducing immunogenic cell death in tumor cells via the release of cytolytic enzymes.^11^ However, such mechanisms upregulating the immune response to tumors also consist of negative feedback loops capable of inducing T cell exhaustion.^12^ In vivo preclinical studies in CT‐26 syngeneic tumor models have shown upregulation of PD‐L1 on tumor cells after administration of fractionated RT, which can then induce T cell exhaustion upon binding to PD‐1 receptors.^13^ Blockade of PD‐L1 during RT has been shown to potentiate the immune response in preclinical tumor models.^14^ RT combined with either sequential or concurrent treatment of anti‐PD‐L1 has been shown to provide potential clinical benefit. In phase III of the PACIFIC clinical trial, concurrent chemoradiotherapy followed sequentially by durvalumab significantly improved both progression‐free survival and overall survival when compared with chemoradiotherapy followed by placebo.^15^, ^16^ However, finding the optimal dosage and schedule of RT in combination with ICB is difficult and is likely to be context dependant.^17^

Inhibitors of the DNA Damage Response Pathway (DDRi) can also aid in the potentiation of the immune response by increasing the sensitivity of tumor cells to RT. DDRis induce additional cell death in the tumor, which further activates cytolytic responses.^18^ Inhibitors of the poly‐ADP‐ribose polymerase (PARP) enzyme or ataxia telangiectasia mutated gene (ATM) have shown efficacy in preclinical tumor models when combined with RT.^19^ However, combining RT with DDRis in the clinic may require further optimization and patient stratification to observe any additional value of these treatment modalities. Attempts to optimize dosage and schedule of these combinations could increase the efficacy to toxicity ratio, and would lead to both prolonged survival as well as improved treatment compliance.^20^ Optimization of dosage regimens to maximize the efficacy to toxicity ratio likely requires rationale developed from preclinical models, as well as mathematical modeling of tumor immune interactions, and how different treatment modalities can potentiate the immune response. Population modeling of RT in combination with anti‐PD‐1 or anti‐PD‐L1 has been previously performed to predict optimal dosage regimens in the context of a highly immunogenic syngeneic tumor model—CT‐26.^21^ However, less immunogenic tumors and dosage optimization of RT in combination with immune checkpoint inhibitors (ICIs) and DDRi‐based therapies are yet to be modeled in this manner. Modeling different tumors with different immunogenicities is crucial to understand differences in treatment responses.

The aim of this study is to describe a mathematical model that incorporates the effects of RT, DDRi, and ICI on the immune system in the context of the colorectal cancer syngeneic tumor model—MC38. Simulations were also performed to explore the role of various components of this model on the response. The model incorporates parameters associated with tumor clearance by T cells, APC death rate, and APC activation of T cells, while fitting for baseline tumor growth rate, APC activation rate due to RTs effects on the tumors proliferative rim, T cell exhaustion, and inhibition of T cell exhaustion by either DDRi or ICI.

## METHODS

### Experimental data

The data for this study consisted of four datasets obtained from the subcutaneous injection of the murine colorectal cancer cell line—MC38, in female C57/BL6 mice. These datasets were derived from time‐to‐event experiments that were designed to assess the efficacy of RT/ICI/DDRi combinations on tumor growth after injection of 10^7^ tumor cells. Mice were euthanized when the tumor reached over 1 cm^3^ or when the tumor was presenting as a wet ulcerated lesion. Mice were also euthanized if welfare criterion or weight‐related criterion were not met during the experiment. All animal procedures were conducted according to UK Home Office guidelines, the Animal Scientific Procedures Act 1986, and protocols were approved by a local animal welfare and ethical review body. The first two datasets (DDRIO1815 and DDRIO1821) were based on experiments that assessed the efficacy of RT in combination with the PARP inhibitor olaparib (PARPi) and/or anti‐mouse‐PD‐L1 (ICI). The other two datasets (DDRIO182 and DDRIO1835) were based on experiments that assessed the efficacy of RT in combination with ATM inhibition using AZD0156 (ATMi) as well as ICI. Treatment and randomization started 3 days after initial injection (day 0). Cohort sizes and treatment regimens are shown in Table 1. For three (DDRIO1815, DDRIO1821, and DDRIO182) of the four datasets, each dataset consisted of 99 mice split over eight cohorts. Fifteen (15) mice were in the control cohort and were given no therapy or mock oral and intraperitoneal treatments (control group and cohort 1). Twelve (12) mice were used in each of the other cohorts. Three cohorts (cohorts 2–4) of 12 mice each were given RT (RT group), DDRi, or ICI as monotherapies, three cohorts (cohorts 5–7) of 12 mice each were given either ICI + DDRi (ICI/DDRi cohort) or RT + DDRi (RT/DDRi cohort) or RT + ICI (RT/ICI cohort), and one final cohort was given all three treatments (RT/DDR/ICI cohort). However, one experiment (DDRIO1835) involving ATM inhibition only had 12 control mice, with six mice per cohort which were treated with RT/ATMi, or RT/ICI. For all mice which were given RT, 10 Gray (Gy) of external beam radiation was given as 2 Gy fractions once per day for 5 days. For mice given anti‐PD‐L1, 10 mg/kg of ICI was given intraperitoneally twice per week on Mondays and Thursdays for 3 weeks, 1–2 h prior to RT. For mice given olaparib (PARPi), 100 mg/kg of olaparib was given orally each day for 21 days. Mice were given the 2.5 mg/kg of ATM inhibitor AZD0156 (ATMi) orally each day for 21 days and both DDRi treatments were given 1–2 h prior to RT. Tumors were measured three times per week until they were euthanized (Monday, Wednesday, and Friday). The full dataset comprised a total of 2143 datapoints. Mice which exhibited a complete response were censored from additional time bins after the complete response was reached.

**TABLE 1:** Description of treatment cohorts for experiments assessing impacts of RT/ICI/DDRi on MC38 tumor growth.

Cohort allocation		Datasets/experiments											
		DDRIO1815 DDRIO1821				DDRIO1824				DDRIO1835			
Cohort	# of mice	Control	RT^a^	ICI^b^	DDRi^c^ (PARPi)	Control	RT^a^	ICI^b^	DDRi^d^ (ATMi)	Control	RT^a^	ICI^b^	DDRi^d^ (ATMi)
1	15/12^e^	✓				✓				✓			
2	12/6^e^		✓				✓				✓		✓
3	12/6^e^			✓				✓			✓	✓	
4^f^	12/6^e^				✓				✓				
5^f^	12/6^e^			✓	✓			✓	✓				
6^f^	12/6^e^		✓		✓		✓		✓				
7^f^	12/6^e^		✓	✓			✓	✓					
8^f^	12/6^e^		✓	✓	✓		✓	✓	✓				

Abbreviations: ATMi, ataxia telangiectasia mutated inhibitor; DDRi, DNA Damage Response Pathway; ICI, immune checkpoint inhibitor; PARPi, PARP inhibitor olaparib; RT, radiotherapy.

^a^ Two Gray (Gy) of external beam RT given once per day from days 0–4.

^b^ Ten mg/kg ICI given twice per week for 3 weeks.

^c^ One hundred mg/kg olaparib is given once per day for 21 days, 1–2 h prior to RT.

^d^ The 2.5 mg/kg AZD0156 given once per day for 21 days 1–2 h prior to RT.

^e^ Number of mice in DDRIO1835.

^f^ Cohorts 4–8 from DDRIO1835 were removed from the analysis prior to fitting due to small cohort sizes (6) representing alternative treatment schedules to those described in the other datasets.

### Model formulation and development

**FIGURE 1:**
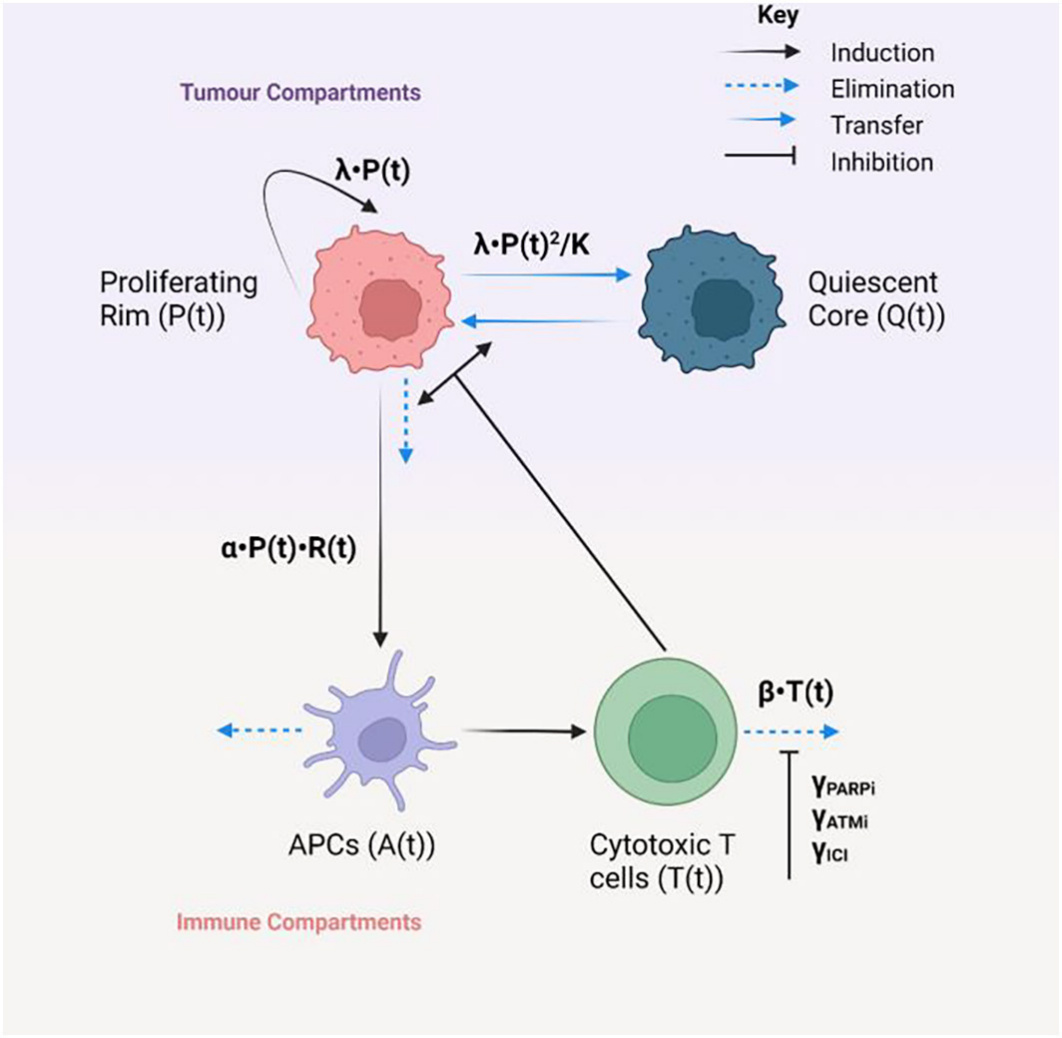
Schematic representation of mathematical model of tumor and immune response fitted to tumor growth data in MC38 syngeneic mouse models. Processes involving fitted parameters are highlighted in bold. Created with BioRender.com. APC, antigen presenting cell.

The mathematical model used to capture tumor‐immune system interactions is shown in Figure 1, model parameters are shown in Table 2. The model incorporates two tumor compartments describing a proliferating rim (*P*(*t*)) and a quiescent core (*Q*(*t*)), as well as two immune compartments describing APCs (*A*(*t*)) and cytolytic T cells *T*(*t*). DAISY was used to confirm that the model was structurally identifiable.^22^ Interindividual variability (IIV) was incorporated into the estimated depth of the quiescent core at day 0 (*Q*_0_), the baseline tumor growth rate (*λ*) and T cell exhaustion rate (*β*). IIV was assumed to be log‐normally distributed and residual unexplained variability was assumed to be additive.

**TABLE 2:** Table of final parameter estimates from model 4 (final model).

Parameter	Description	Units	Value (RSE%)	Citation
*P*_0_	Initial rim depth	mm	2.4	36
*Q*_0_	Initial core depth	mm	3.83 (2)	Fitted
*A*_0_	Initial APC concentration	cells mm^−1^	0	Fixed^a^
*C*_0_	Initial active CD8 concentration	cells mm^−1^	0	Fixed^a^
*K*	Rim carrying capacity	mm	2.4	36
*K*_PT_	CD8 rim killing rate	cell^−1^ day^−1^	0.001	21
*K*_PTQ_	CD8 transfer of core to rim	cell^−1^ day^−1^	0.001	21
*K*_R_	Transfer affinity from the core.	mm	0.1	Fixed^b^
*K*_ϕA_	Baseline influx of APCS	cells day^−1^ mm^−1^	0.001	Fixed^a^
*K*_Aϕ_	APC natural death rate	day^−1^	0.648	37
*K*_AT_	APC activation of CD8 cells	day^−1^	9.12	37
*α*	APC recruitment by RT	cells Gy^−1^ mm^−1^ day^−1^	3.06 (2)	Fitted
*λ*	Baseline logistic rim growth rate	day^−1^	0.239 (3)	Fitted
*β*	Natural CD8 exhaustion rate	day^−1^	0.278 (23)	Fitted
*η*(*Q*_0_)	IIV in *Q*_0_	mm^2^	0.0429 (18)	Fitted
*η*(*λ*)	IIV in *λ*	day^−2^	0.064 (17)	Fitted
*η*(*β*)	IIV in *β*	day^−2^	1.25 (20)	Fitted
COR(*Q*_0_, *λ*)	Correlation between IIV estimates for *Q*_0_ and *λ*	Dimensionless	−0.27	Fitted
COR(*Q*_0_, *β*)	Correlation between IIV estimates for *Q*_0_ and *β*	Dimensionless	−0.04	Fitted
COR(*λ*, *β*)	Correlation between IIV estimates for *λ* and *β*	Dimensionless	−0.11	Fitted
*γ*_ATMi_	ATMi inhibition of exhaustion	Dimensionless	4.34 (40)	Fitted
*γ*_PARPi_	PARPi inhibition of exhaustion	Dimensionless	2.03 (46)	Fitted
*γ*_ICI_	ICI inhibition of exhaustion	Dimensionless	11.9 (32)	Fitted
*σ*	Additive RV	mm^2^	0.506 (4)	Fitted

Abbreviations: APC, antigen presenting cell; ATMi, ataxia telangiectasia mutated inhibitor; ICI, immune checkpoint inhibitor; IIV, interindividual variability; PARPi, PARP inhibitor olaparib; RSE%, percentage relative standard error; RT, radiotherapy; RV, random unexplained variability.

^a^ Assumed that minimal/no active CD8 cells are present without administration of RT.

^b^ Assumed for consistency with the rate which CD8^+^ T cells remove cells in the rim.

### The tumor compartments

*P*(*t*) is assumed to grow logistically, where competition for space within the rim leads to the transfer of cells in the rim and into *Q*(*t*). Cells within the rim can be targeted by *T*(*t*), the system of equations is set up so that for approximately every cell killed in the rim by *T*(*t*), one cell from the core is transferred into the rim as the tumor shrinks, and that the amount of cells within the tumor is proportional to the tumor diameter. For all assessed models, the tumor compartments are unchanged (Equation 1).

(1) $$\begin{array}{c} \overset{\text{Proliferative}\;\mathrm{rim}\;\text{growth rate}}{\overbrace{\frac{dP\left(t\right)}{dt}}}=\overset{\text{Logistic growth}}{\overbrace{\lambda \cdot P\left(t\right)\cdot \left(1-\frac{P\left(t\right)}{K}\right)}}+\overset{Q\;\text{to}\;P\;\text{transfer}}{\overbrace{\frac{K_{\mathrm{PTQ}}\cdot P\left(t\right)\cdot T\left(t\right)\cdot Q\left(t\right)}{K_{R}+Q\left(t\right)}}}-\overset{\mathrm{CD}8\;\text{targeted}\;\mathrm{rim}\;\text{death}}{\overbrace{K_{\mathrm{PT}}\cdot P\left(t\right)\cdot T\left(t\right)}}\\ \;\overset{\text{Core growth rate}}{\overbrace{\frac{dQ\left(t\right)}{dt}}}=\overset{\text{Competition inside}\;\mathrm{rim}}{\overbrace{\frac{\lambda \cdot P{\left(t\right)}^{2}}{K}}}-\overset{Q\;\text{to}\;P\;\text{transfer}}{\overbrace{\frac{K_{\mathrm{PTQ}}\cdot P\left(t\right)\cdot T\left(t\right)\cdot Q\left(t\right)}{K_{R}+Q\left(t\right)}}} \end{array}$$

where K, λ, $K_{R}$, $K_{\mathrm{PT}}$ and $\;K_{\mathrm{PTQ}}$ are the rim carrying capacity, the baseline tumor growth rate, the affinity of transfer from the core to the rim, the rate of T cell mediated death within the rim, and the rate of T cell mediated transfer of the core into the rim, respectively.

### The dendritic cell compartment

Two compartments in this model describe the immune system, *A*(*t*) represents APCs that react to the cell death that occurs in the rim due to RT (*R*(*t*)) where *R*(*t*) is a Heaviside function active between days 0 and 4, and the Heaviside amplitude is scaled to represent the daily dose of radiation in Gray (Gy). *A*(*t*) is responsible for the activation of cytolytic tumor specific CD8 cells (*T*(*t*)). This model assumes comparatively little baseline influx of *A*(*t*) and *T*(*t*) prior to RT. For all models, the *A*(*t*) compartment remains unchanged (Equation 2).

$$\begin{array}{c} \;\overset{\mathrm{APC}\;\text{influx rate}}{\overbrace{\frac{dA\left(t\right)}{dt}}}=\overset{\text{Baseline influx}}{\overbrace{K_{\mathrm{\phi A}}}}+\overset{R\left(t\right)\;\text{and}\;P\left(t\right)\;\text{induced recruitment}}{\overbrace{\alpha \cdot P\left(t\right)\cdot R\left(t\right)}}\\ \;-\overset{\text{Natural}\;\mathrm{APC}\;\text{death}}{\overbrace{K_{\mathrm{A\phi}}\cdot A}\left(t\right)\;} \end{array}$$

(2) $$R\left(t\right)=2\;\mathrm{Gy}\cdot \text{Heaviside}\left(4.1-t\right)$$

where $K_{\mathrm{\phi A}}$, α and $K_{\mathrm{A\phi}}$ are the baseline APC recruitment rate, RT mediated APC recruitment rate, and APC natural death rate, respectively.

### The T cell compartment

For all models, *T*(*t*) is assumed to be recruited by *A*(*t*), whereas *T*(*t*) naturally decays (exhaustion) at rate *β*. For the first model (model 1), the exhaustion rate is unchanged by the incorporation of either DDRi or ICI.

(model 1) $$\begin{array}{c} \overset{T\;\text{cell influx rate}}{\overbrace{\frac{dT\left(t\right)}{dt}}}=\overset{\mathrm{APC}\;\text{activation of}\;T\;\text{cells}}{\overbrace{K_{\mathrm{AT}\cdot }\cdot A\left(t\right)}}-\overset{T\;\text{cell exhaustion}\;}{\overbrace{\beta \cdot T\left(t\right)}} \end{array}$$

where $K_{\mathrm{AT}}$ and β are the APC mediated T cell activation rate and T cell exhaustion rate, respectively.

Due to the dosing regimen for both DDRi and ICI leading to complete saturation of inhibitory compounds.^23^, ^24^, ^25^, ^26^ Furthermore, as all experiments used the same doses of PARPi, ATMi, and ICI, parameterizing the model as a pharmacokinetic‐pharmacodynamic (PK‐PD) model is more difficult and would not be particularly informative. Taken together, there was little benefit in involving the effects of PKs in this model, and the PDs can be modeled simply as inhibition constants.

For model 2, DDRi is assumed to have no effect on T cell exhaustion, and any combination effects are assumed to be dependent on the presence of ICI only. ICI is assumed to inhibit the rate of T cell exhaustion by a factor of 1 + $\gamma_{\mathrm{ICI}}$, where $\gamma_{\mathrm{ICI}}$ is a parameter that describes the impact of ICI on T cell exhaustion at the given dosage and schedule.

(model 2) $$\begin{array}{c} \overset{T\;\text{cell influx rate}}{\overbrace{\frac{dT\left(t\right)}{dt}}}=\overset{\mathrm{APC}\;\text{activation of}\;T\;\text{cells}}{\overbrace{K_{\mathrm{AT}}\cdot A\left(t\right)}}-\overset{T\;\text{cell exhaustion}}{\overbrace{\frac{\beta \cdot T\left(t\right)}{1+\gamma_{\mathrm{ICI}}}}} \end{array}$$

For model 3, DDRi is also assumed to have an impact on T cell exhaustion, but there is no significant difference between PARPi and ATMi in inducing this effect. The effects of combination therapy are assumed to inhibit the exhaustion rate by a factor of 1 + $\gamma_{\mathrm{ICI}}$ + $\gamma_{\text{DDRi}}$, where $\gamma_{\text{DDRi}}$ is a parameter which describes the impact of either DDRi on the T cell exhaustion rate.

(model 3) $$\begin{array}{c} \overset{T\;\text{cell influx rate}}{\overbrace{\frac{dT\left(t\right)}{dt}}}=\overset{\mathrm{APC}\;\text{activation of}\;T\;\text{cells}}{\overbrace{K_{\mathrm{AT}}\cdot A}\left(t\right)}-\overset{T\;\text{cell exhaustion}}{\overbrace{\frac{\beta \cdot T\left(t\right)}{1+\gamma_{\mathrm{ICI}}+\gamma_{\text{DDRi}}}}} \end{array}$$

For the full model (model 4), the effects of PARPi and ATMi are assumed to be sufficiently different and inhibit the rate of T cell exhaustion by $\gamma_{\text{PARPi}}$ and *γ*_ATMi_, respectively. This differential impact on PARPi and ATMi is assumed to extend to RT/DDRi/ICI combinations.

(model 4) $$\begin{array}{c} \overset{T\;\text{cell influx rate}}{\overbrace{\frac{dT\left(t\right)}{dt}}}=\overset{\mathrm{APC}\;\text{activation of}\;T\;\text{cells}}{\overbrace{K_{\mathrm{AT}}\cdot A}\left(t\right)}-\overset{T\;\text{cell exhaustion}}{\overbrace{\frac{\beta \cdot T\left(t\right)}{1+\gamma_{\mathrm{ICI}}+\gamma_{\text{PARPi}}+\gamma_{\text{ATMi}}}}} \end{array}$$

where $\gamma_{\text{PARPi}}$ and $\gamma_{\text{ATMi}}$ are parameters describing the differential impacts of incorporating PARPi and ATMi with RT or RT/ICI on the T cell exhaustion rate.

Tests for significant improvements in model fit for models 1, 2, and the full model were performed using the likelihood ratio test (LRT). Model 3 was compared with model 4 using the Akaike Information Criterion (AIC) due to the lack of nesting between these models.

### Model fitting and simulation

A list of model parameters, definitions, values, and sources are shown in Table 2. The model was fitted simultaneously to control, RT, RT/DDRi, RT/ICI, and RT/DDRi/ICI cohorts with NONMEM version 7.4.3 using Stochastic Approximation Expectation Maximization (SAEM). Fitting with the first order conditional estimation (FOCE) method was used to obtain an appropriate initial estimate. For the final parameter estimates, expectation only importance sampling was used to calculate log‐likelihood values, followed by a final covariance step to obtain standard error estimates. The parameters which were estimated using FOCE‐SAEM were *Q*_0_, *λ*, *α*, and *β*, *γ*_PARPi_, *γ*_ATMi_, and *γ*_ICI_ as well as IIV on *Q*_0_, *λ*, and *β*. IIV was represented as a full block matrix in ℝ^3×3^. Random unexplained variability (RV) was assumed to be additive. The NONMEM model code is supplied within the supplementary material (Code S1, Data S1). Convergence was checked by use of five sequential parallel estimations with 10% increments in the fixed effect parameters, as well as a further five parallel estimations where the initial estimates of the fixed effect parameters were set to 0.33, 0.67, 1, 1.5, and 3 × the final parameter estimates shown in Table 2. There were 95% of expected values for a given parameter $(\theta_{95,i}$) that were calculated using the typical value and IIV parameters, as shown in Equation 3.

(3) $$\theta_{95,i}=\theta_{i}\cdot \exp\left(\pm 1.96\cdot \omega_{i}\right)$$

where $\theta_{i}$ represents the typical value output of a given parameter *I*, and $\omega_{i}^{2}$ is the expected IIV of the corresponding parameter.

Individual fits, diagnostics, and visual predictive checks (VPCs) were evaluated using NONMEM and graphs plotted with R version 3.6.3.^27^ Heat maps showing simulation end points were plotted in R. The full model consists of 25 parameters, 14 of which were fitted, including IIV and RV. Simulations of alternative potencies was also performed to assess the effect of alternative potential dosing regimens on the relative efficacy of RT/ICI compared with corresponding tri‐therapies (Methods S1).

## RESULTS

### Model parameter estimates

Final parameter estimates of the model are listed in Table 2 with percentage relative standard errors (RSE%) for each estimated parameter. LRT and AIC indicated that the full model (model 4) significantly improved model fit compared to the reduced models (Table 3). Model 1 was confirmed to be structurally globally identifiable (Methods S1 and Code S2). Parameter estimates indicated a *Q*_0_ of 3.83 mm. When combined with the initial rim depth, this suggests an estimated initial tumor diameter of 6.23 mm, which agrees with measured mean diameter (6.31 mm). Assessment of RSE% values suggested highly precise estimates for *Q*_0_, *λ*, and *α*. However, estimates were not as precise for *γ*_PARPi_ and *γ*_ATMi_, with RSE% values of 41% and 46%, respectively. These RSE% estimates were still considered to be within acceptable limits to confirm the beneficial impact of incorporating DDRi in combination with RT.

**TABLE 3:** Comparison of −2LL_IMP_ and AIC estimates from models 1–4.

Model	T cell exhaustion rate	Description	*N* of parameter	−2LL_IMP_	AIC
1	*β*	Combinations have no impact on T cell exhaustion	11	2005	2027
2	*β*/(1 + *γ*_ICI_)	ICI reduces T cell exhaustion	12	1962	1986
3	*β*/(1 + *γ*_ICI_ + *γ*_DDRi_)	ICI and DDRi reduces T cell exhaustion	13	1949	1975
4	*β*/(1 + *γ*_ICI_ + *γ*_PARPi_ + *γ*_ATMi_)	ICI, PARPi, and ATMi reduces T cell exhaustion	14	1942	1970

Abbreviations: AIC, Akaike Information Criterion; ATMi, ataxia telangiectasia mutated inhibitor; DDRi, DNA Damage Response Pathway; ICI, immune checkpoint inhibitor; LL, log‐likelihood; PARPi, PARP inhibitor olaparib.

When assessing convergence from varied initial conditions, parameters *Q*_0_, *λ*, *α*, and *β* typically converged to values within 20% of the final parameter estimates shown in Table 3, however, the final estimates of *γ*_PARPi_, *γ*_ATMi_, and *γ*_ICI_ were more sensitive to varied initial estimates (Tables S1 and S2; Figures S1 and S2). The population T cell exhaustion rate for control and RT treated cohorts was estimated to be 0.278 day^−1^ (*T*½ = 2.49 days). Ninety‐five percent of the expected exhaustion rates are between 0.03 and 2.48 day^−1^ in the context of RT as a monotherapy. In the context of bi‐therapies, 95% of PARPi mediated inhibition of therapy is suggested to induce an exhaustion rate of between 0.009 and 0.81 day^−1^ (typical value 0.09 day^−1^). ATMi is expected to have a larger inhibitory effect on T cell exhaustion, where 95% of the exhaustion rates are expected to be within 0.005 and 0.464 day^−1^ (typical value 0.05 day^−1^). ICI, however, is suggested to have the largest impact on T cell exhaustion, reducing the overall exhaustion rate by 93%. RV is estimated to be ~0.5 mm^2^.

### Model diagnostics

**FIGURE 2:**
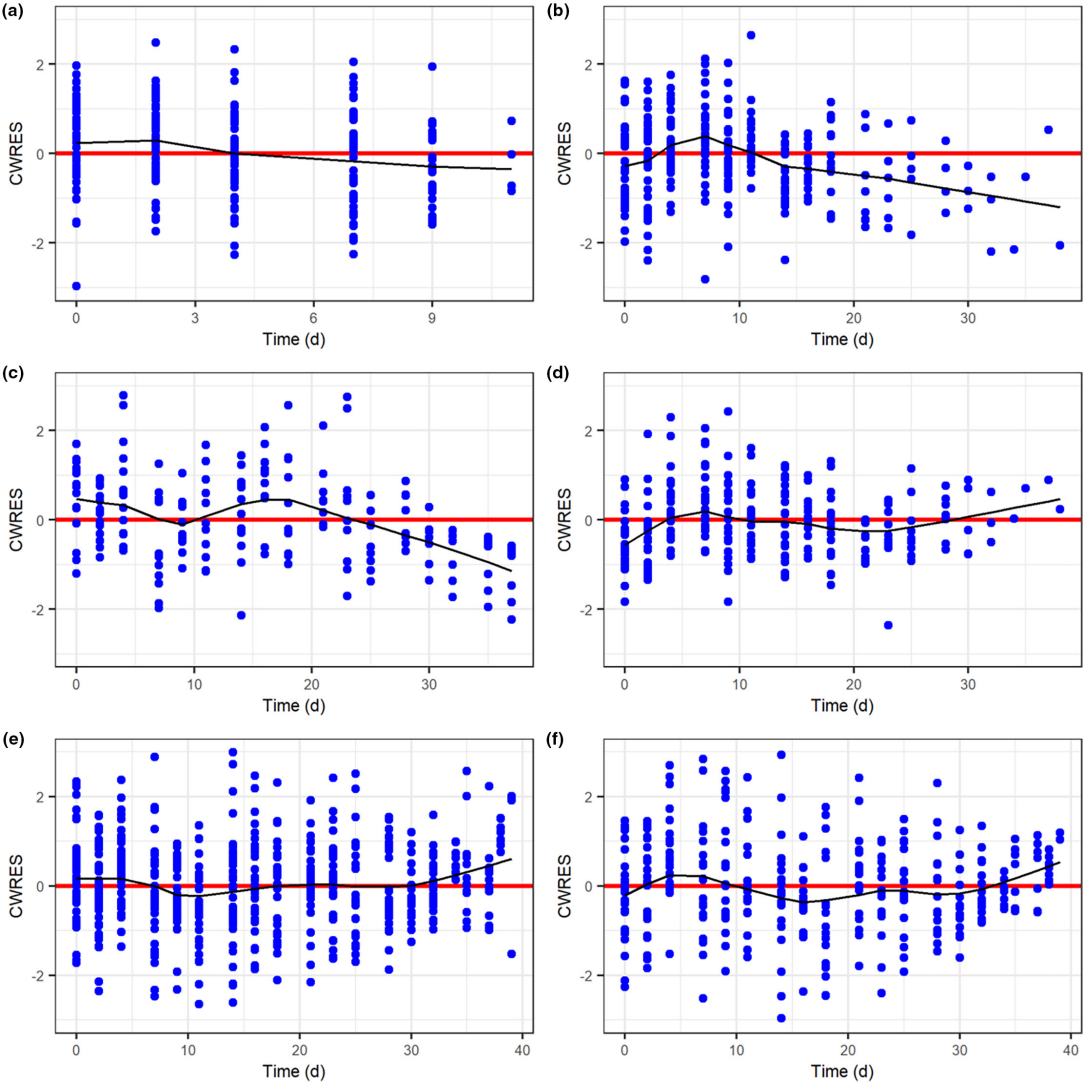
Diagnostic CWRES plots vs time for model 4. (a) Control cohorts, (b) RT treated cohorts, (c) RT/ATMi treated cohorts, (d) RT/PARPi treated cohorts, (e) RT/ICI cohorts. (f) RT/DDRi/ICI treated cohorts. The annotation is reflective of the mean and variance of the CWRES. Black solid lines represent loess regression lines accounting for 33% of neighboring points within a local region. ATMi, ataxia telangiectasia mutated inhibitor; CWRES, conditional weighted residual; DDRi, DNA Damage Response Pathway; PARPi, PARP inhibitor olaparib; RT, radiotherapy.

**FIGURE 3:**
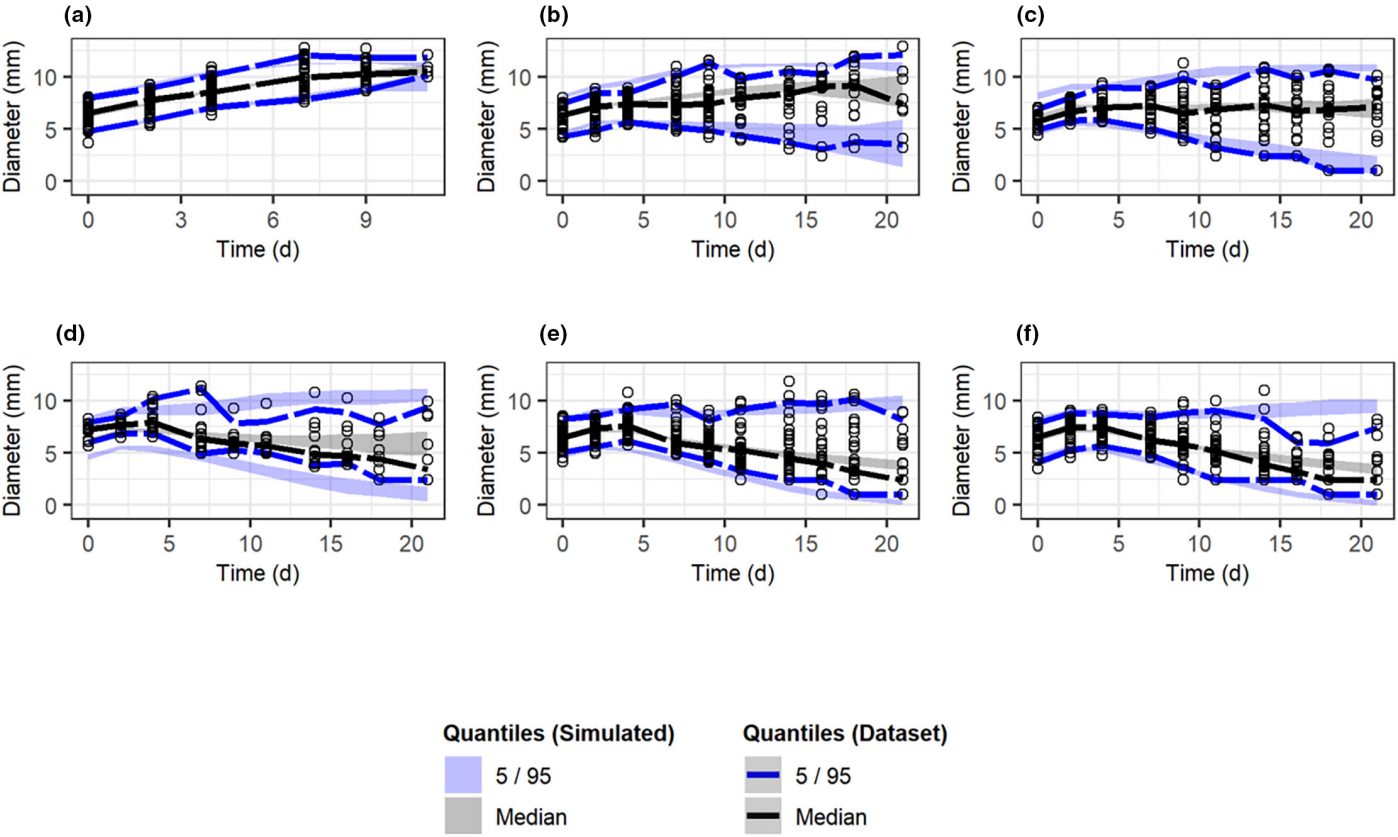
VPC of model 4 stratified by treatment. The 95% confidence intervals of the 5% and 95% quantile values of tumor sizes from simulated datasets (shaded regions) overlaid with corresponding quantiles from the observed dataset. Plotted points show the observed data. (a) Control cohorts, (b) RT treated cohorts, (c) RT/PARPi treated cohorts, (d) RT/ATMi treated cohorts, (e) RT/ICI treated cohorts, (f) RT/DDRi/ICI treated cohorts. ATMi, ataxia telangiectasia mutated inhibitor; DDRi, DNA Damage Response Pathway; ICI, immune checkpoint inhibitor; PARPi, PARP inhibitor olaparib; RT, radiotherapy; VPC, visual predictive check.

Conditional weighted residuals from the full model were calculated from individual fits and plotted against time. Due to the extensive amount of data, plots of residuals against time were split between the different treated cohorts (Figure 2). Whereas plots suggested relatively evenly spread residuals, after day 18 there are signs of bias in some of the treated cohorts. Loess regression lines indicate that RT treated cohort tumor sizes are overestimated after day 18 (Figure 2b). This time‐related bias is more apparent in the RT/ATMi treated cohort (Figure 2c). RT/PARPi, RT/ICI, and RT/PARPi/ICI cohorts show a relatively even distribution of residuals over time, with a moderate underestimation of tumor sizes at later timepoints (Figure 2d–f). Additional diagnostic plots indicated that population predictions over‐estimate the expected tumor size, particularly diameters below the limit of quantification as well as diameters close to the expected end point. Observed diameters from RT/ICI treated cohorts appear to be underestimated by the population prediction, whereas observed diameters from RT treated cohorts appear to be overestimated by the population prediction (Figure 3a,b). Individual predicted diameters were reasonably well estimated against the observed diameter (Figure 3c,d).

### Graphical model diagnostic with VPC

One thousand simulations were performed in NONMEM using the parameter values in Table 2 and plots were generated in R to obtain the 5, 50, and 95 percentiles for tumor diameters of each simulated dataset (Figure 3). These values were overlaid with the observed percentile values of tumor diameters from the data. To simulate the impact of dropout, only simulated tumor diameters of 11.44 mm or smaller were included in the VPCs, this corresponds to tumors which reach 1.5 cm^3^. As expected, control tumor data from all four studies were well captured with this model (Figure 3a), as significant overlap is observed between the percentile values from the control dataset, and the corresponding percentile regions in the simulated dataset. VPCs indicated that the upper, median, and lower quantiles produced from the simulated dataset parameter values followed similar trends as the observed upper and lower quantile estimates (Figure 3b–f). However, with respect to stronger treatment cohorts, such as RT/ATMi, RT/ICI, and RT/PARPi/ICI, VPCs indicated that model parameters overestimated tumor sizes at later timepoints.

### Simulation of alternative drug potencies

The results from the VPCs indicated that at the given dosage and schedule, the impacts of RT/ICI were too strong to observe a significant improvement in response in RT/DDRi/ICI cohorts. A comparison of the upper, median, and lower quantile estimates between RT/ICI and RT/DDRi/ICI cohorts show significant overlap in expected growth trajectories, whereas RT/PARPi and RT/ATMi simulations show significant differences in median percentile estimates to RT/DDRi/ICI. This indicates that the dosage of RT/ICI at the current schedule could be reduced, and dose reduction of ICI may also lead to observed significant benefits of RT/DDRi/ICI which are comparable to the current RT/ICI schedule.

Due to the large dosages given relative to the half‐maximal inhibitory concentration for ATMi and dissociation constant for ICI,^23^, ^24^ an integrated PK‐PD model would provide little information on how to optimize dosing strategies, as the high doses given mean that any variability observed is unlikely to be due to variability in the PK profiles of these drugs. In addition, the lack of alternative dosage regimens of DDRi increases the difficulty in developing an extensive dose response curve which would be more informative when assessing the effects of RT/DDRi. Considering these limitations of the experimental design, the parameter estimates produced in Table 2 that describe the impacts of ATMi and ICI were considered the maximum effects observable with these treatments, and simulations were performed assuming lower parameter estimates for *γ*_ICI_ and *γ*_ATMi_, to mimic the effect of theoretical dosages and schedules producing different changes in active T cell profiles (Methods S1).

**FIGURE 4:**
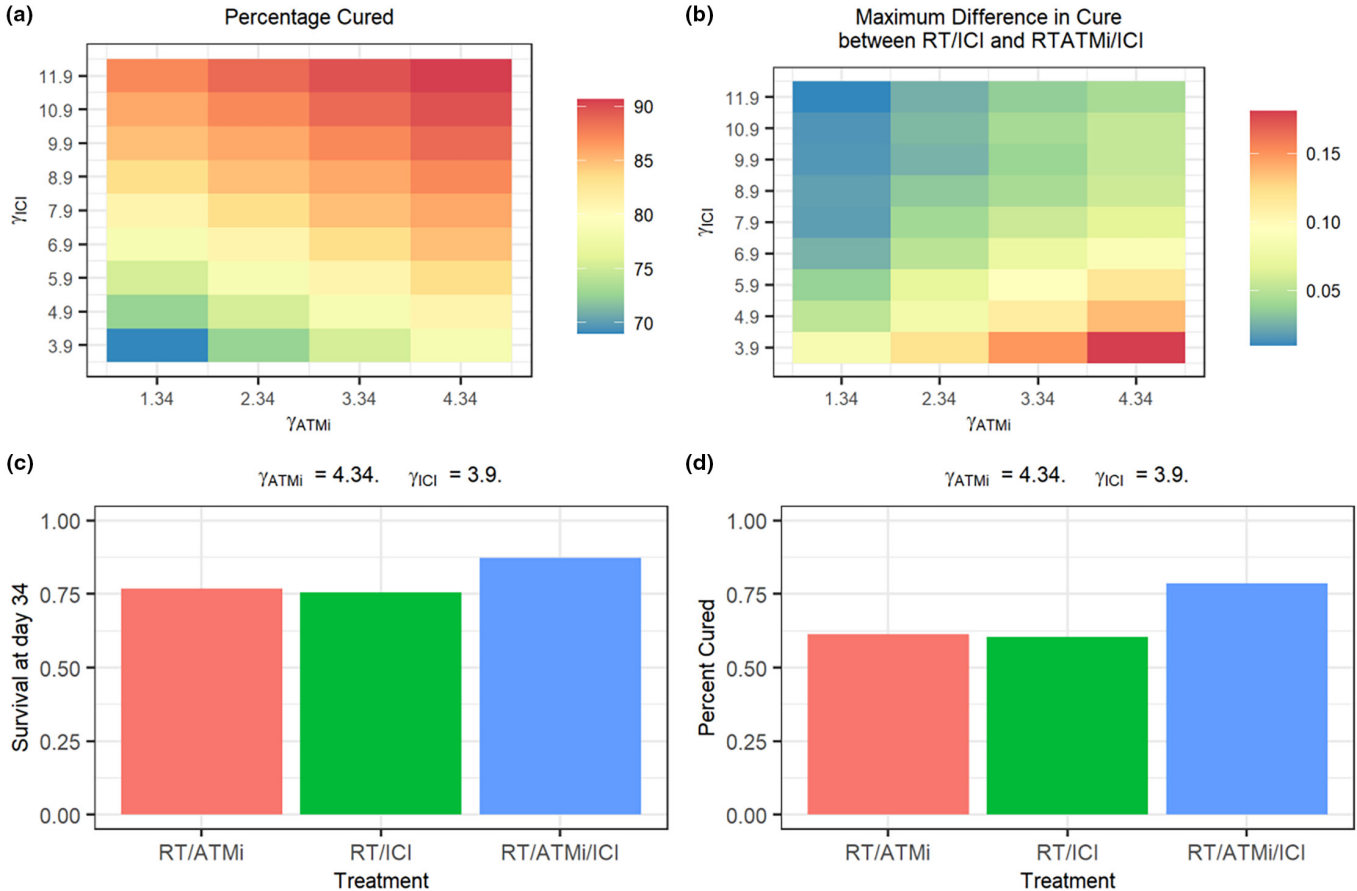
Simulations with modified potency values for *γ*_ATMi_ and *γ*_ICI_. (a) Percentage of mice cured at day 34 when combining alternative *γ*_ATMi_ and *γ*_ICI_ potencies. (b) Differences in cure rates between RT/ICI and RT/ATMi/ICI. (c) Relative survival at day 34 in RT/ICI, RT/ATMi, and RT/ATMi/ICI cohorts when *γ*_ATMi_ = 4.34 and *γ*_ICI_ = 3.9. (d) Cure rates observed when *γ*_ATMi_ = 4.34 and *γ*_ICI_ = 3.9. ATMi, ataxia telangiectasia mutated inhibitor; ICI, immune checkpoint inhibitor; RT, radiotherapy.

The results of the simulations suggested that the impact of high doses of ICI can lead to issues in proving the effects of both ATMi and ICI for high efficacy tri‐therapy. Simulations suggested that the maximum difference in cure rate between bi‐therapy and tri‐therapy occurred when, *γ*_ICI_ = 3.9, and *γ*_ATMi_ = 4.34 (Figure 4b). This coincides with the 2.5 mg/kg dose of AZD0156 for 5 days, and doses which lead to 32% of the maximal effect observed during RT/ICI. This leads to a cure rate of ~60% in bi‐therapy cohorts (Figure 4d), which increases to ~79% in the tri‐therapy cohort, which is a similar value to the cure rates simulated after RT/ICI bi‐therapy (86%) at the dosage used in the above experiments.

## DISCUSSION

Mixed‐effects modeling of tumor growth in response to RT and combinations of RT/ICI/DDRi therapies is still in relatively early phases, with few models of RT/ICI combinations that are robust enough to provide suitable information when translating results to the clinic. This study describes initial phases of model development which incorporate tumor growth, T cell mediated immunogenic cell death, APC activation by RT, APC mediated T cell activation, as well as T cell exhaustion. Model parameters RSE% were shown to be sufficiently precise, the least precise parameters being parameters associated with DDRi impacts on T cell exhaustion, which may be partially due to fewer data points in the RT/PARPi and RT/ATMi treated cohorts, variability in relative efficacy between studies, as well as the redundancy of adding DDRi to the RT/ICI dosage regimen. Although this model has currently only been assessed in MC38 tumors, its structural properties may be well‐suited to describe other preclinical and clinical datasets.

Model diagnostics indicated a time‐dependent bias in RT and RT/ATMi treated cohorts at later timepoints. Reasons for this may be due to limited data available at later timepoints in these cohorts as mice drop out of the study due to reaching the expected end point. With respect to RT/ATMi, this bias is partially due to the lower sample size (18 mice) compared to other regimens (24–48 mice), as well as a larger amount of inter‐study variability. One study with a cohort of 12 mice exhibited cure in seven of 12 mice when given RT/ATMi therapy, whereas the other study with six mice exhibited no cures.

When assessing convergence from varied initial conditions, parameters *Q*_0_, *λ*, *α*, and *β* converged to values typically within 20% of the final parameter estimates observed in Table 2, indicating robust estimation of these parameters. However, the final estimates of *γ*_PARPi_, *γ*_ATMi_, and *γ*_ICI_ were more sensitive to varied initial estimates. This sensitivity is likely due to the similar tumor trajectories observed between RT/ICI and corresponding tri‐therapies, making individual potency parameter estimates which extend to the tri‐therapy regimens difficult to capture appropriately.

The VPCs effectively captured how tumors responded to the majority of dosage regimens, with RT/ATMi bi‐therapy showing the least consistency between experimental data and simulated values, likely due to reasons mentioned above. VPCs indicated that in stronger dosing regimens, such as RT/ATMi, RT/ICI, and tri‐therapy regimens, the model simulations over estimate tumor size at later timepoints. This moderate underestimation of the response could be due to the impact of mice dropping after the tumor reaching the end point volume of 1 cm^3^. This leads to a reduced observed median tumor size at later timepoints. This highlights the necessity for robust experiments with prolonged end points, or the need to modify the dataset to incorporate a dropout effect more effectively. Studies assessing different DDRi dosage regimens also may have alleviated these issues, as it would have been useful for model parameterization and development of adequate dose response curves.

It is difficult to underpin why there are differences between studies that lead to different cure rates, and reproducibility has been reported as a major issue in biological experiments.^28^ This highlights the need for performing in vivo experiments in a repeatable manner to minimize the biological variability. Additional data, such as cage cleaning cycles, should also be recorded, as the microbiome is also known to affect the tumor response to various drugs.^29^ Additional attempts to make experiments more repeatable would also involve ensuring that tumor sizes are similar between experiments at the time of randomization^30^ and potentially use cell line samples which have undergone a sufficiently similar amount of passages.^31^

The effect of RT is relatively consistent between studies, which explains why the RSE% for parameters associated with APC recruitment and T cell exhaustion are highly precise and why the VPCs capture the RT monotherapy effects well. Taken together, these findings justify the large cohort sizes in each experiment, which gives extensive data points for more improved fitting with less residual error and better internal validation. Incorporation of additional drugs to a patient's treatment regimen provide an avenue to not only impact additional targets within a heterogeneous tumor, but also give potential to minimize the side effects of drugs given at high doses. Both model fits, VPCs, and optimization simulations suggested that the effects of DDRi are overshadowed by the near maximal effect of ICI in these experiments. There are indeed results within the literature that demonstrate improved efficacy of ATR inhibitors (ATRi) in combination with RT and ICI. Sheng et al.^32^ were able to show significant efficacy of RT/ATRi/ICI due to improved immunogenicity and tumor control after three doses of 6Gy external beam radiation in the hepatocellular carcinoma syngeneic model—Hepa 1–6. However, the varying immunophenotypic backgrounds between Hepa 1–6 and MC38 are likely to influence the results and explain some of the differences in efficacy between different RT/DDRi/ICI. For example, Hepa 1–6 tumors have a larger percentage of CD45^+^ cells prior to treatment compared with MC38 and a larger abundance of CD8^+^ T cells, whereas MC38 also has a larger proportion of M1 associated macrophages.^33^

For each of the experimental studies used during model fitting, high doses of ICI and DDRi were given relative to their respective dissociation constants and target affinities.^23^, ^24^ Consequently, addition of PK parameters would provide little beneficial information in this model. To conceptualize what the impacts of alternative dosing regimen may look like, simulations were performed while varying the values of *γ*_ATMi_ and *γ*_ICI_. The effects of ATMi were simulated instead of PARPi due to the expected improved efficacy of RT/ATMi compared with RT/PARPi at their given dosages and schedules. Because of this higher efficacy, simulations would indicate a larger relative difference in efficacy between tri‐therapies and bi‐therapies, while also maintaining similar efficacies observed during RT/ICI with 10 mg/kg anti PD‐L1. This can also be observed when assessing simulations of *γ*_ATMi_ at 2.34 units, which is a similar value to the expected potency of PARPi. Simulations at the above potency indicate that higher doses of ICI would show lower relative differences in efficacy between tri‐therapy and the corresponding bi‐therapy.

Simulations indicated that modification of the dosage regimen, leading to a 68% reduction in potency of ICI relative to the potency observed in the above experiments, in combination with the current ATMi dosage regimen, would provide the largest difference in cure rates between the bi‐therapy regimens and corresponding tri‐therapies. Simulations indicated that these potency values could lead to a cure rate of 79%, which is comparable to the cure rates simulated during RT/ICI high dose bi‐therapy. Doses which provide this level of potency could then be informative for preclinical PD studies, in order to elucidate the mechanisms which RT/DDRi/ICI can combine to synergistically improve local tumor control. Preclinical experiments now can be performed with the aim of finding the ideal ICI dosage regimen in combination with RT that leads to a 32% improvement in population T cell exhaustion rates compared with RT alone, which would also serve to test the hypothesis which the above simulations have indicated, as the model specification relies on the assumption that the dose‐efficacy relationship for RT/DDRi/ICI is linear, of which there are limited data available to confirm or deny this.

The model developed above contains IIV captured in both baseline tumor characteristics as well as IIV in expected T cell exhaustion rates, however, other models of RT in combination with ICI have negative feedback loops incorporated into the model,^21^ which this model lacks. Syngeneic models vary drastically in immunophenotype,^34^ and consequently, the mechanisms which can lead to negative feedback also may have the propensity to vary depending on the tumor model chosen,^13^, ^18^ or the dosage schedule.^35^ T cell mediated negative feedback could be incorporated into the model with an additional compartment which is upregulated by T cells, where T cell exhaustion is dependent on this additional compartment. Additional work will now be carried out which will assess which biomarkers may be relevant to describe the differential effects of RT in combination with DDRi or ICI.

In summary, this report describes a successful development of a model which incorporates tumor growth via a proliferating rim and quiescent core, as well as dendritic cell recruitment and T cell activation, to populations of mice given RT/DDRi/ICI combination therapy. The model concludes that the effects of RT/ICI are near maximal and do not allow for significant improvement in efficacy by DDRi. Additional simulations suggest that reduction of the ICI efficacy by ~68% will lead to a larger effect observed between RT/ICI and RT/ATMi/ICI. Further preclinical experiments can now be produced in order to test this hypothesis and validate the above findings. The next stages of this work will be to analyze experimental data to assess whether additional biomarkers and negative feedback loops can be incorporated into the model, as this could provide additional evidence for alternative dosage regimen being beneficial to improve the rates of cure.

## AUTHOR CONTRIBUTIONS

D.H., K.O., and S.G. wrote the manuscript. K.O., J.Y., H.M., L.A., S.G., and M.D. designed the research. D.H., P.F., A.S., and E.C. performed the research. D.H. analyzed the data.

## FUNDING INFORMATION

This work was funded through the Biotechnology and Biosciences Research Council (BBSRC) Industrial CASE studentship in collaboration with AstraZeneca (AZ), UK (Grant BB/T508512/1).

## CONFLICT OF INTEREST STATEMENT

S.G., M.D., A.S., P.F., and E.C. are AstraZeneca employees and hold AstraZeneca shares and stock options. J.Y. is a former AstraZeneca employee. All other authors declared no competing interests for this work.

## Supporting information


Data S1

Supplementary Code S1

Supplementary Code S2

Data S2
