# Supplementary material for: Mixed effects modeling of radiotherapy in combination with immune checkpoint blockade or inhibitors of the DNA damage response pathway
Source: CPT Pharmacometrics Syst Pharmacol. 2023 Sep 18;12(11):1640–52. doi: 10.1002/psp4.13026 (PMC10681475; doi:10.1002/psp4.13026)
Supplement: Supplementary file 2 — Supplementary Code S1 [file PSP4-12-1640-s001.docx]

**Supplemental code S1. Model code for use with corresponding simulated dataset in NONMEM**

$PROBLEM MC38 RT/DDRi/ICI MODEL

$INPUT ID TIME TRT DV STUDY

$SUBROUTINE ADVAN13 TRANS1 TOL=6

$MODEL NCOMPARTMENTS=4

$DATA 2531_3_supp_111431_rxh2m5.csv

$PK

Q0 = THETA(1)*EXP(ETA(1))

LAMBDA = THETA(2)*EXP(ETA(2))

ALPHA = THETA(3)

BETA = THETA(4)*EXP(ETA(3))

GAMMA = THETA(5)

DELTA = THETA(6)

ZETA = THETA(7)

;Model parameters

KDTL = 0.027*24

KDL = 0.027*24

KDLCD8 = 0.38*24

KCD8lt = 0.06

;tumour ics

A_0(1) = 2.4

A_0(2) = Q0

;A(t) ics

A_0(3) = 0

;T(t) ics

;A_0(4) = 0

$DES

HEAV1 = 0

RT1 = 0

RT2 = 0

PARP = 0

ATM1 = 0

IO1 = 0

ATM2 = 0

IO2 = 0

IF (TIME.LT.4) HEAV1 = 1

IF (TIME.LT.1.1) HEAV2 = 1

IF (TRT.GT.1) RT1 = 1 ;All cohorts given RT

IF (TRT.EQ.3.OR.TRT.EQ.6) PARP = 1 ; RT/PARPi and RT/PARPi/ICI cohorts respectively

IF (TRT.EQ.4.5.OR.TRT.EQ.7) ATM1 = 1 ; RT/ATMi and RT/ATMi/ICI cohorts respectively

IF (TRT.EQ.5.5.OR.TRT.EQ.6.OR.TRT.EQ.7) IO1 = 1 RT/ICI and Tritherapy cohorts.

;tumour compartments

FQ = A(2)/(0.1 + A(2))

DADT(1) = (LAMBDA)*A(1)*(1 - A(1)/(2.4)) + 0.001*A(4)*A(1)*FQ - 0.001*A(4)*A(1)

DADT(2) = ((LAMBDA)*A(1)**2)/(2.4) - 0.001*A(4)*A(1)*FQ

;dendritic cell compartments

DADT(3) = 0.001 + ALPHA*A(1)*(HEAV1*RT1*2) - KDTL*A(3)

;pdl1 compartment solved

;cd8 cell comparmtents

DADT(4) = KDLCD8*A(3) - BETA*A(4)/(1 + GAMMA*PARP + DELTA*ATM1 + ZETA*IO1)

$ERROR

Y = (A(1) + A(2)) + EPS(1)

;estimation based theta initial values

$THETA (0,5,10)

(0,0.19)

(0,3,5)

(0,0.16)

(0,1,100)

(0,1,100)

(0,1,100)

$OMEGA BLOCK(3)

0.0475

-0.0184 0.0547

0.01 -0.01 2

$SIGMA 0.6

$ESTIMATION METHOD=1 PRINT=100 MAXEVAL=9999

$ESTIMATION METHOD=SAEM NITER=500 AUTO=1 PRINT=50

$ESTIMATION METHOD=IMP EONLY=1 PRINT=1 NITER=5 ISAMPLE=1000 MAPITER=0

$COVARIANCE SLOW
