# Supplementary material for: Mixed effects modeling of radiotherapy in combination with immune checkpoint blockade or inhibitors of the DNA damage response pathway
Source: CPT Pharmacometrics Syst Pharmacol. 2023 Sep 18;12(11):1640–52. doi: 10.1002/psp4.13026 (PMC10681475; doi:10.1002/psp4.13026)
Supplement: Supplementary file 3 — Supplementary Code S2 [file PSP4-12-1640-s002.docx]

**Supplemental code S2. Model code for use in DAISY.**

WRITE “A FOUR STATE MODEL”$

%B_ IS THE VARIABLE VECTOR

B_:={P,Q,A,Ti,Ti2,Ti3,y1}$

FOR EACH EL_ IN B_ DO DEPEND EL_,T$

%B1_ IS THE UNKNOWN PARAMETER VECTOR

B1_:={lm,al,be,gam1,gam2}$

%NUMBER OF STATES

NX_:=4$

%NUMBER OF INPUTS

NU_: =0$

%NUMBER OF OUTPUTS

NY_:=1$

%MODEL EQUATIONS

C_:={df(P,t) = lm*P*(1-P/2.4) + 0.001*P*(Ti)*Q/(0.1 + Q) – 0.001*P*(Ti),

df(Q,t) = lm*P*P/2.4 – 0.001*P*Q*(Ti)/(0.1 + Q),
df(A,t) = 0.001 + al*P – 0.648*A,
df(Ti,t) = 9.12*A – be*Ti,
y1 = (P+Q)}$

FLAG_:=1$

SEED_:=25$

DAISY()$

%KNOWN INITIAL CONDITIONS

ICK_:={P=2.4,A=0,Ti=0}

%UNKNOWN ICS

ICUNK_:={Q=Qo}$

CONDINIZ()$

END$
