# Supplementary material for: Mixed effects modeling of radiotherapy in combination with immune checkpoint blockade or inhibitors of the DNA damage response pathway
Source: CPT Pharmacometrics Syst Pharmacol. 2023 Sep 18;12(11):1640–52. doi: 10.1002/psp4.13026 (PMC10681475; doi:10.1002/psp4.13026)
Supplement: Supplementary file 4 — Data S2 [file PSP4-12-1640-s004.docx]

**Supplemental material.**

**Supplemental methods.**

**Confirmation of model identifiability.**

To test whether the model was structurally identifiable, DAISY (Differential Algebra for Identifiability of Systems) was used. The initial reduced model (model 1) was considered the base model to analyse for identifiability, as the extended models are mathematical analogues of the reduced model, applied to different treatment cohorts. Briefly, the model developed is assessed for identifiability by transformation into a characteristic set of equations referred to as the “Characteristic set of a differential ideal”. More information can be found in the corresponding citation in the main text.

**Parameter estimation settings.**

For the final model, initial estimations were run through a first order conditional estimation step with no interaction, with up to 9999 maximum evaluations. The final estimates of the FOCE were then run through 500 iterations of the SAEM algorithm. An additional option (AUTO=1) was supplemented into the SAEM algorithm to allow NONMEM to determine the optimal number of chains used by the Metropolis-Hastings algorithm. After 500 iterations of SAEM, the final estimates were run through 5 iterations of expectation only (EONLY) importance sampling. With 1000 random samples per subject used (ISAMPLE=1000). The first iteration was set to rely on conditional means and variances from the final SAEM estimation (MAPITER=0). The final estimates of the importance sampling step were then run through a slow computation of covariance to estimate relative standard errors. The NONMEM model code can be seen below (See supplementary code).

**Simulation of alternative potencies.**

In order to effectively characterise the percentage of responders in each cohort, modification of the current dataset was performed to ensure that every individual mouse had tumour diameter data at and up until day 34. In the context of survival, any individual subject simulation where the diameter had not yet reached 10mm by day 34 was considered a survivor. In the context of cure, any individual subject simulation exhibiting diameter values below the detection limit (2.4mm) at day 34 were considered cured mice. Different candidate dosing regimens were produced by modifying the potency parameter estimates for ICI and ATMi and for each candidate dosing regimen, 100 simulations of 12 mice in each treatment cohort were conducted in NONMEM. A total of 36 candidate potencies were simulated, by simulating γ_ICI_ values between 3.9 and 11.9 in increments of 1, as well as simulating γ_ATMi_ values between 1.34 and 4.34 in increments of 1. All combinations of each γ_ICI_ and γ_ATMi_ values within these ranges were simulated. The aims of the simulations were to characterise each dosing regimen by both rates of survival and rates of cure, and to find an optimal dosing regimen for RT/ATMi/ICI. The potency combination which maximised the difference in survival and cure rates between the candidate RT/ICI dosing regimen and the candidate RT/ATMi/ICI dosing regimen was considered the optimal potency.

**Figure S1. Convergence plots of the SAEM Objective Function Value (OFV) across 500 iterations (Stochastic/Accumulation mode) shown in Supplementary table 1. A) Initial estimate X1, B) Initial estimate X1.1, C) Initial estimate X1.2, D) Initial estimate X1.3, E) Initial estimate X1.4, F) Initial estimate X1.5,**

**A)**

**
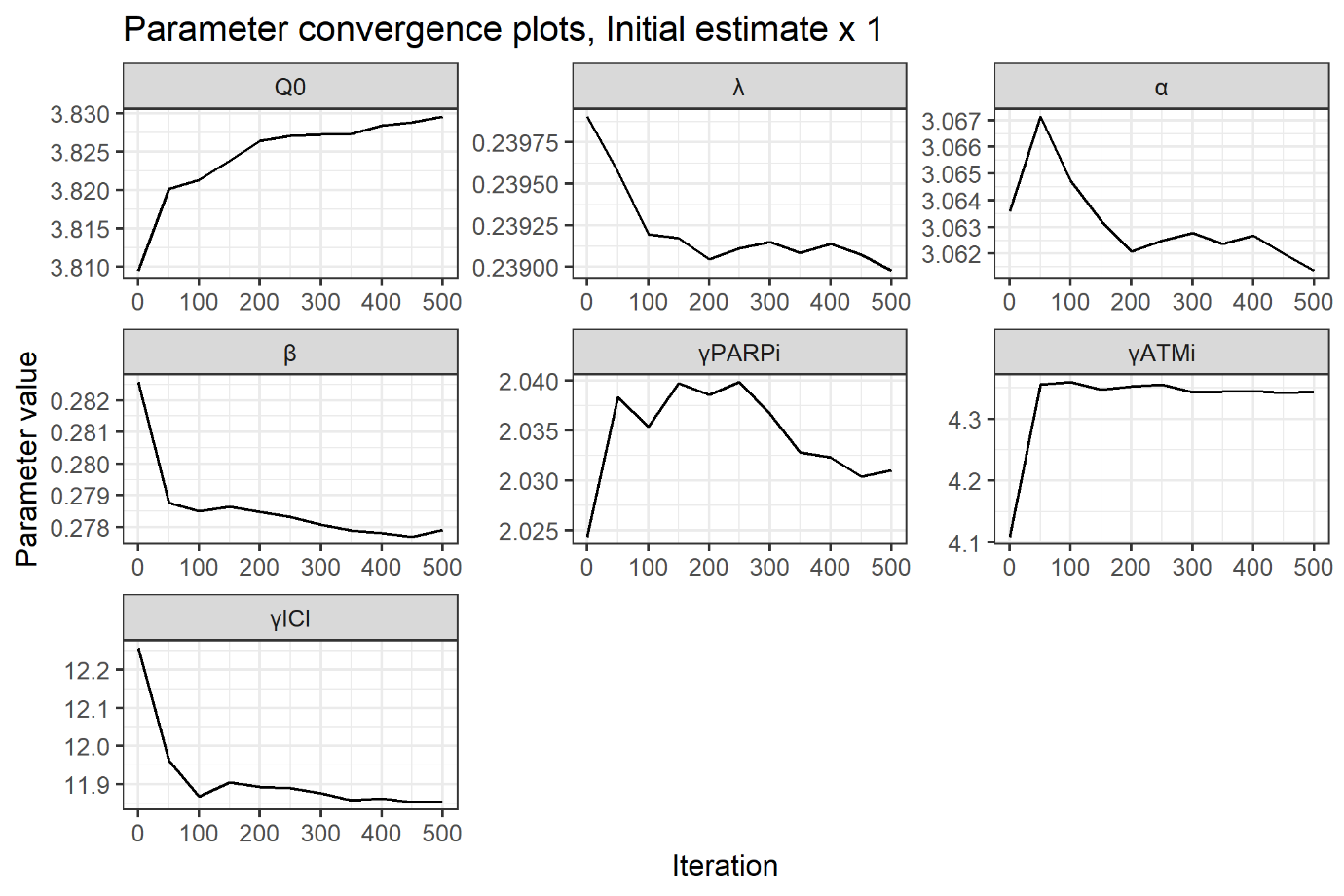
**

**B)**

**
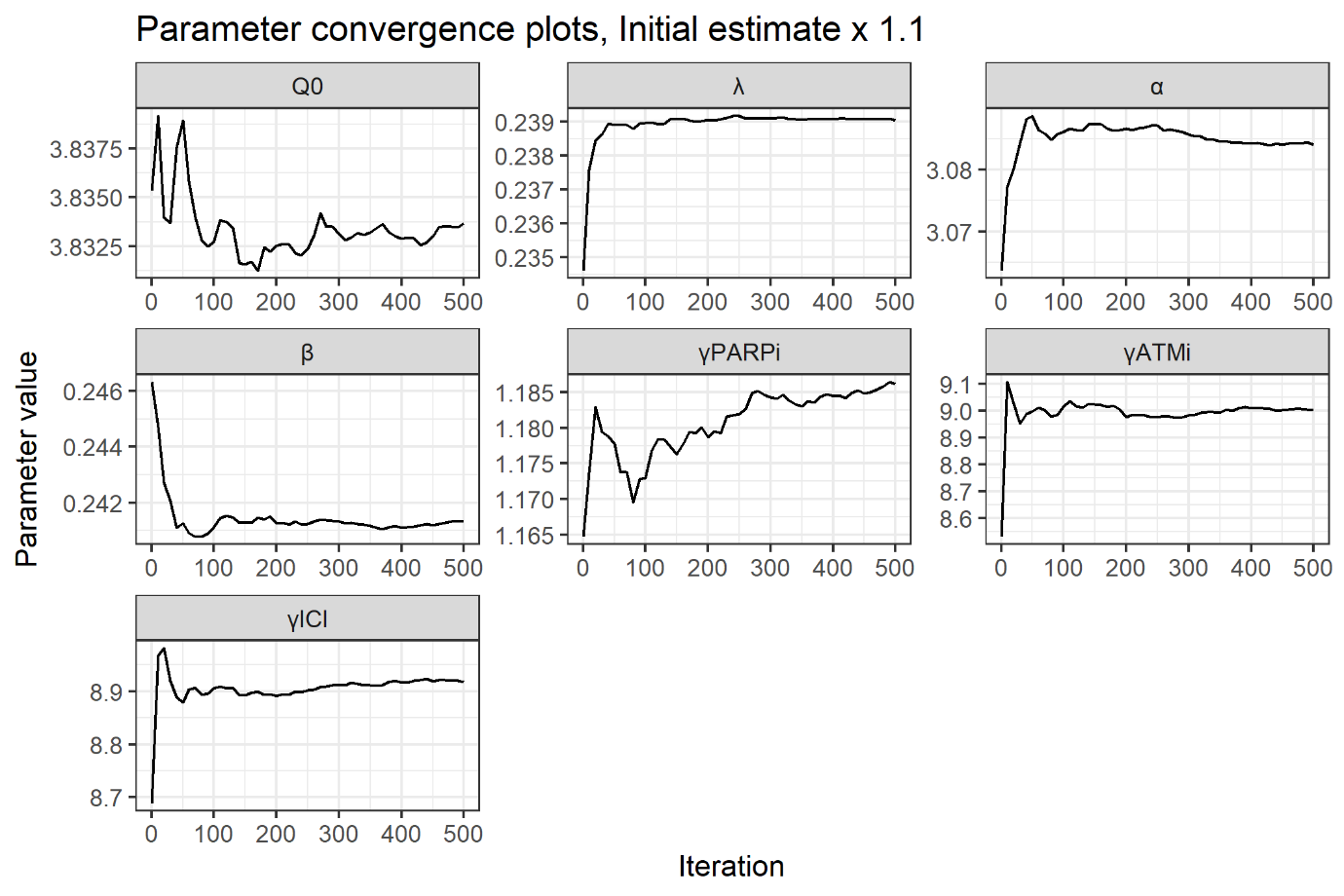
**

**C)**

**
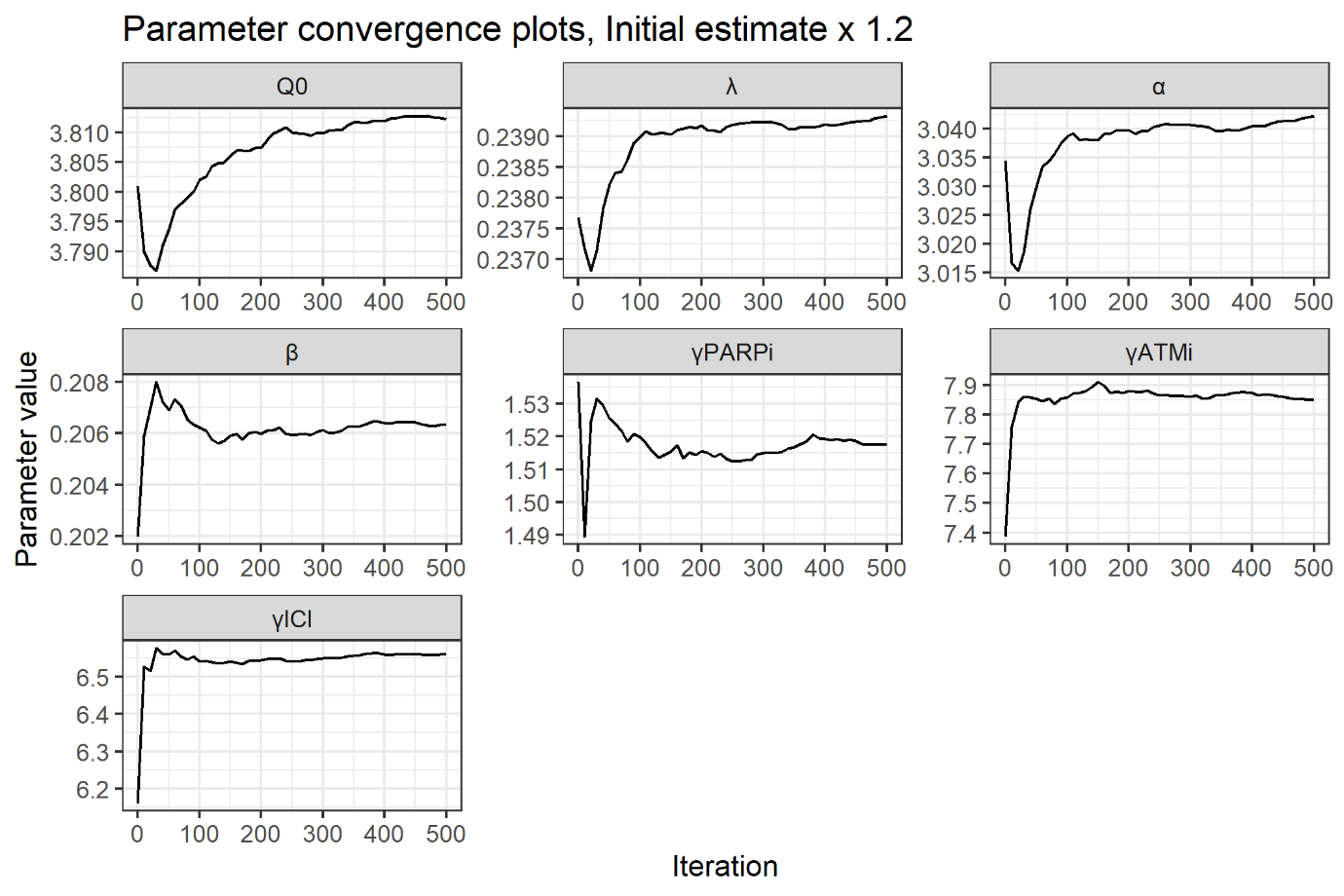
**

**D)**

**
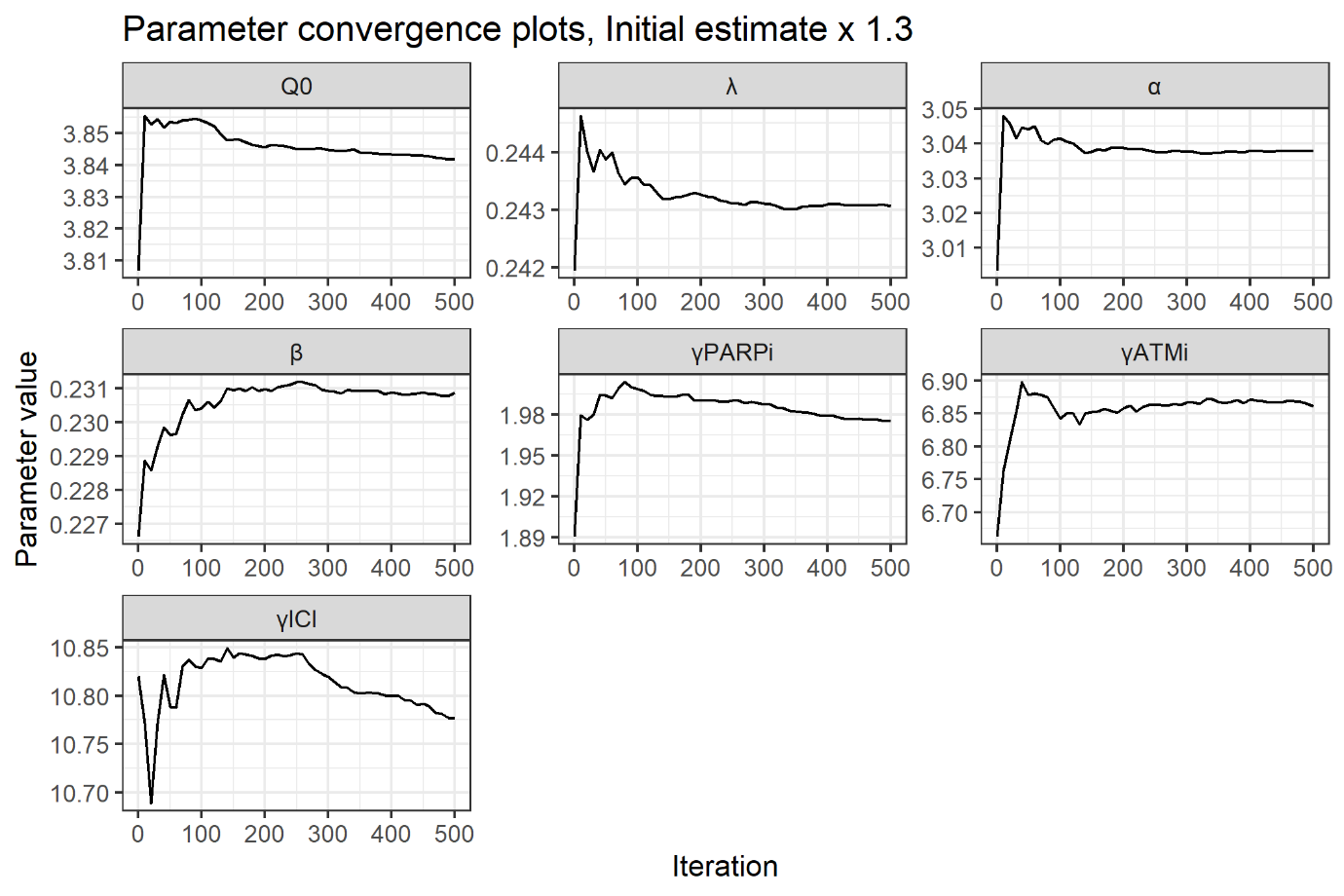
**

**E)**

**
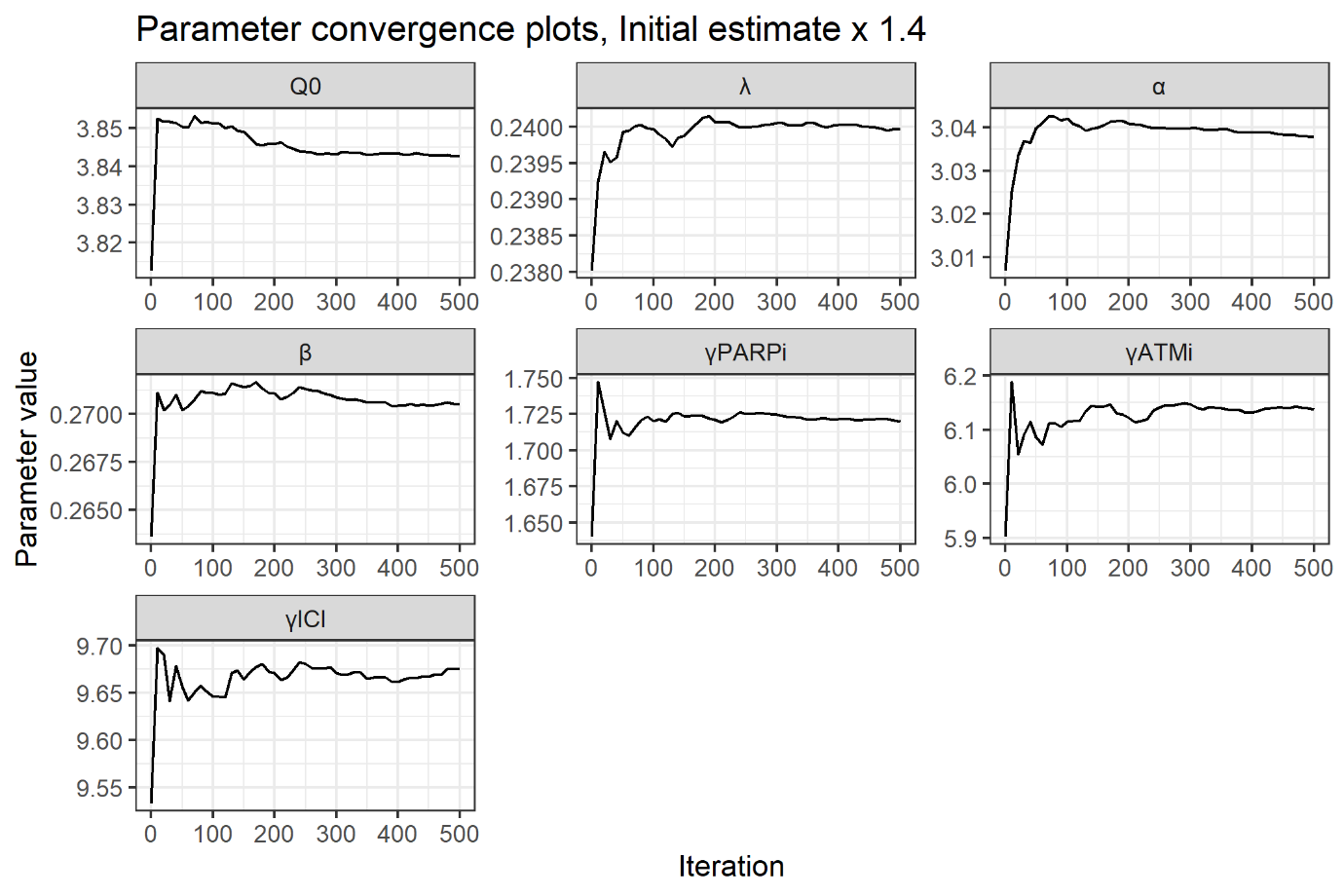
**

**F)**


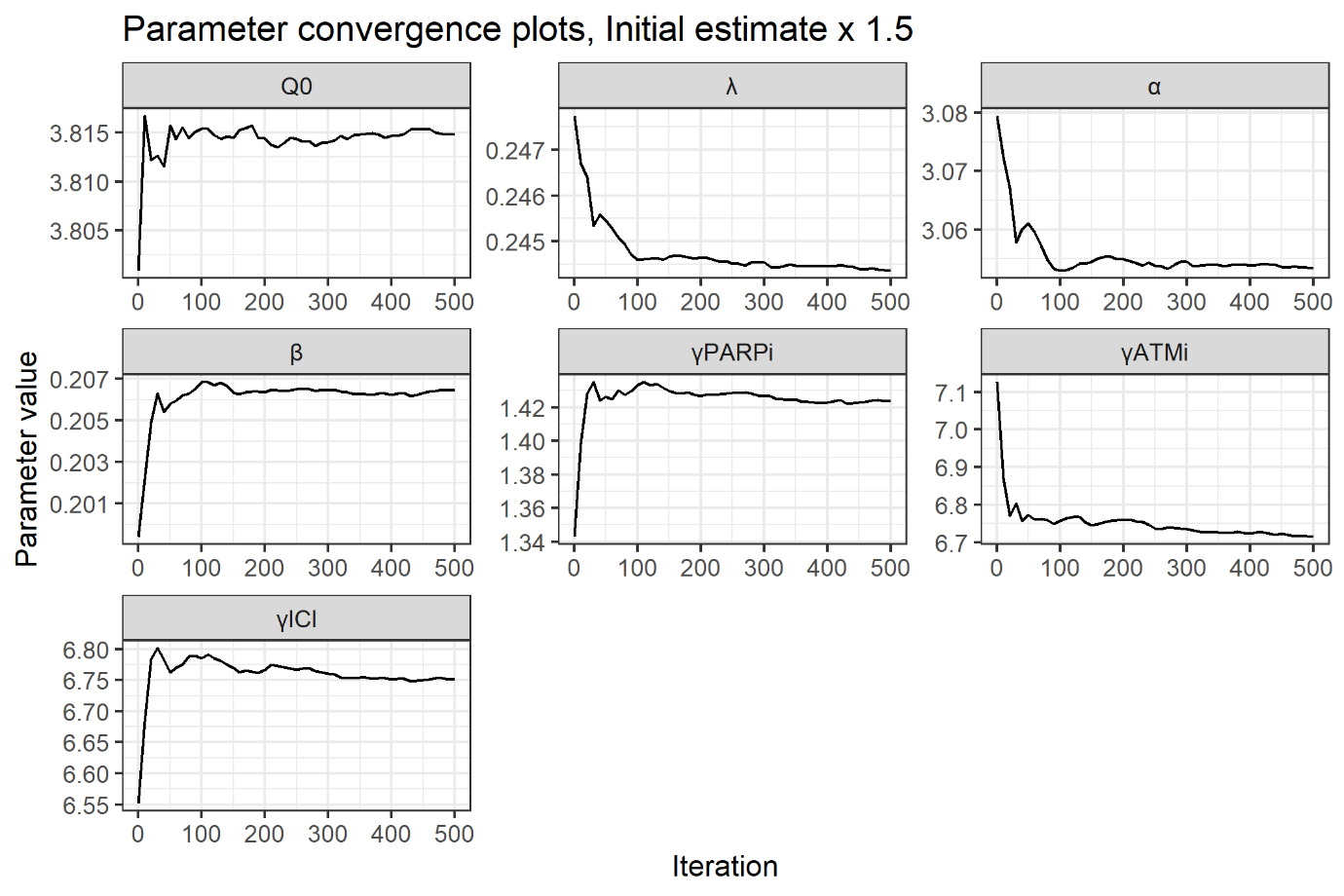


**Figure S2. Convergence plots of the SAEM Objective Function Value (OFV) across 500 iterations (Stochastic/Accumulation mode) shown in Supplementary table 2. A) Final estimate X1, B) Final estimate X0.33, C) Final estimate X0.67, D) Final estimate X1.5, E) Final estimate X3.**

**A)**


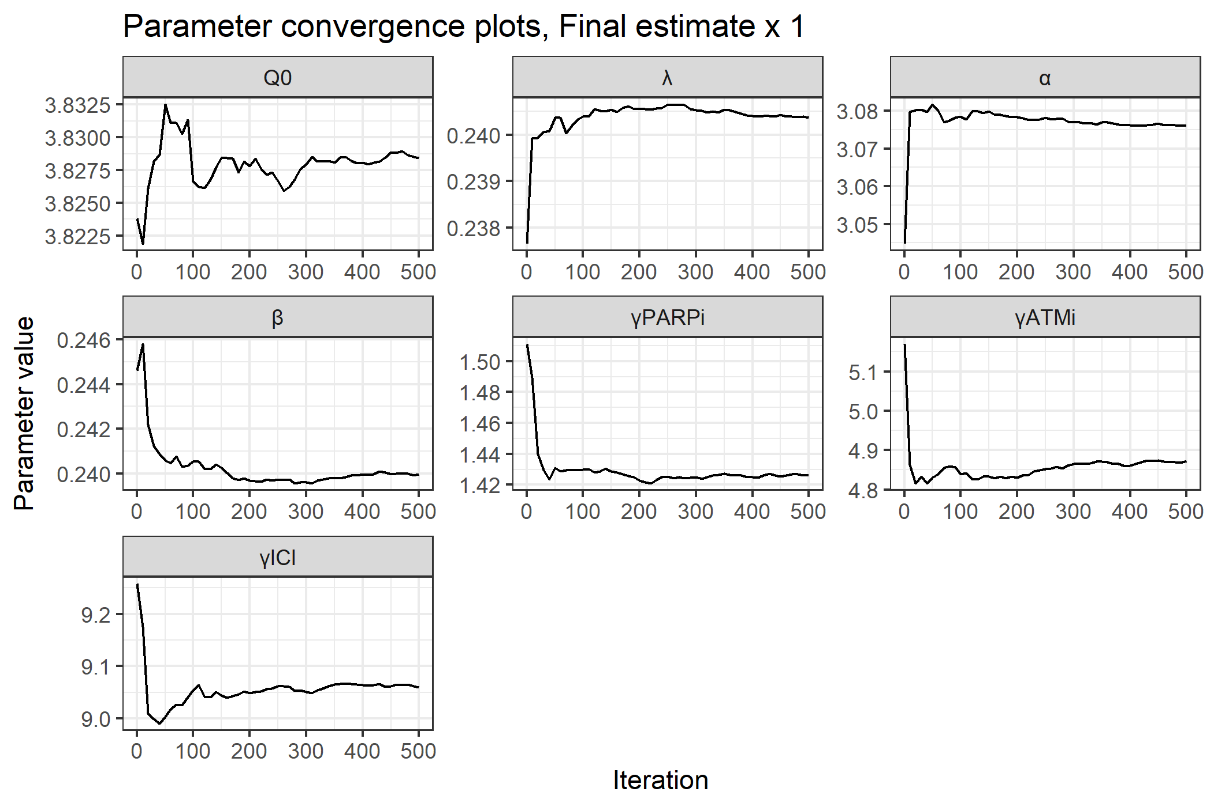


**B)**

**
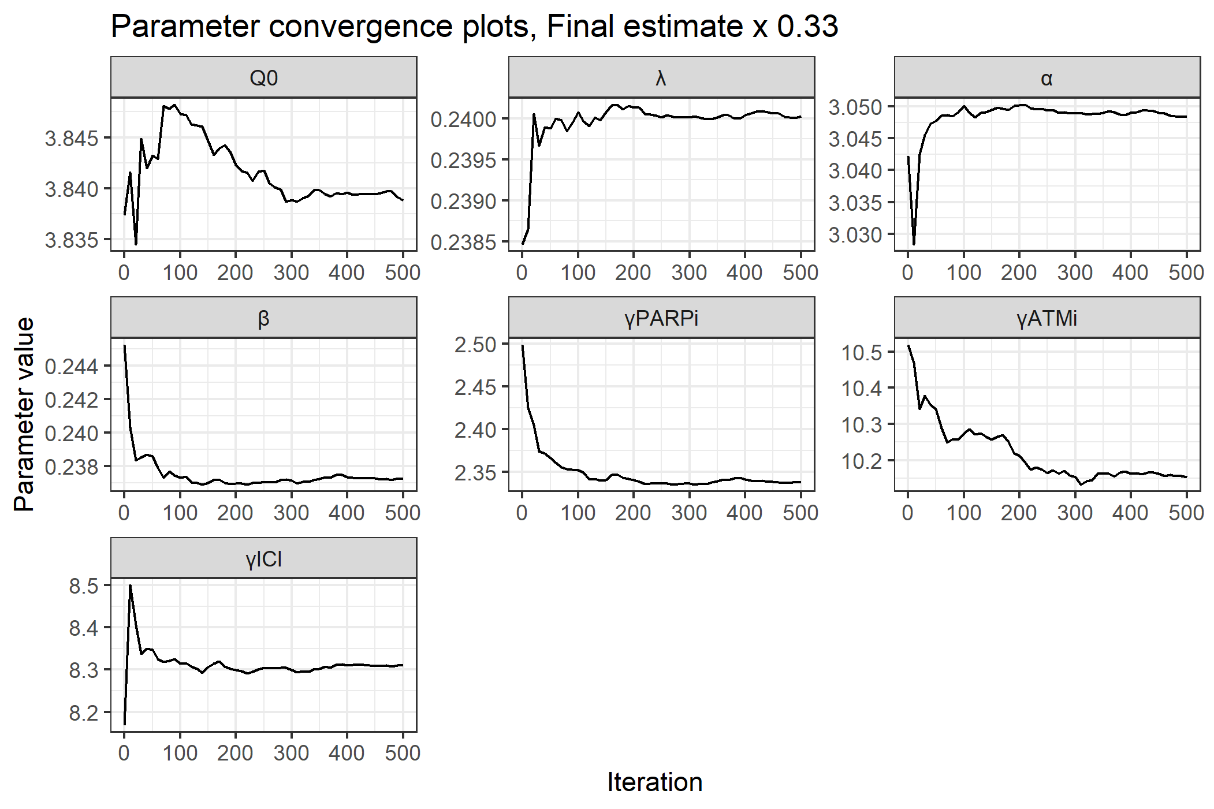
**

**C)**

**
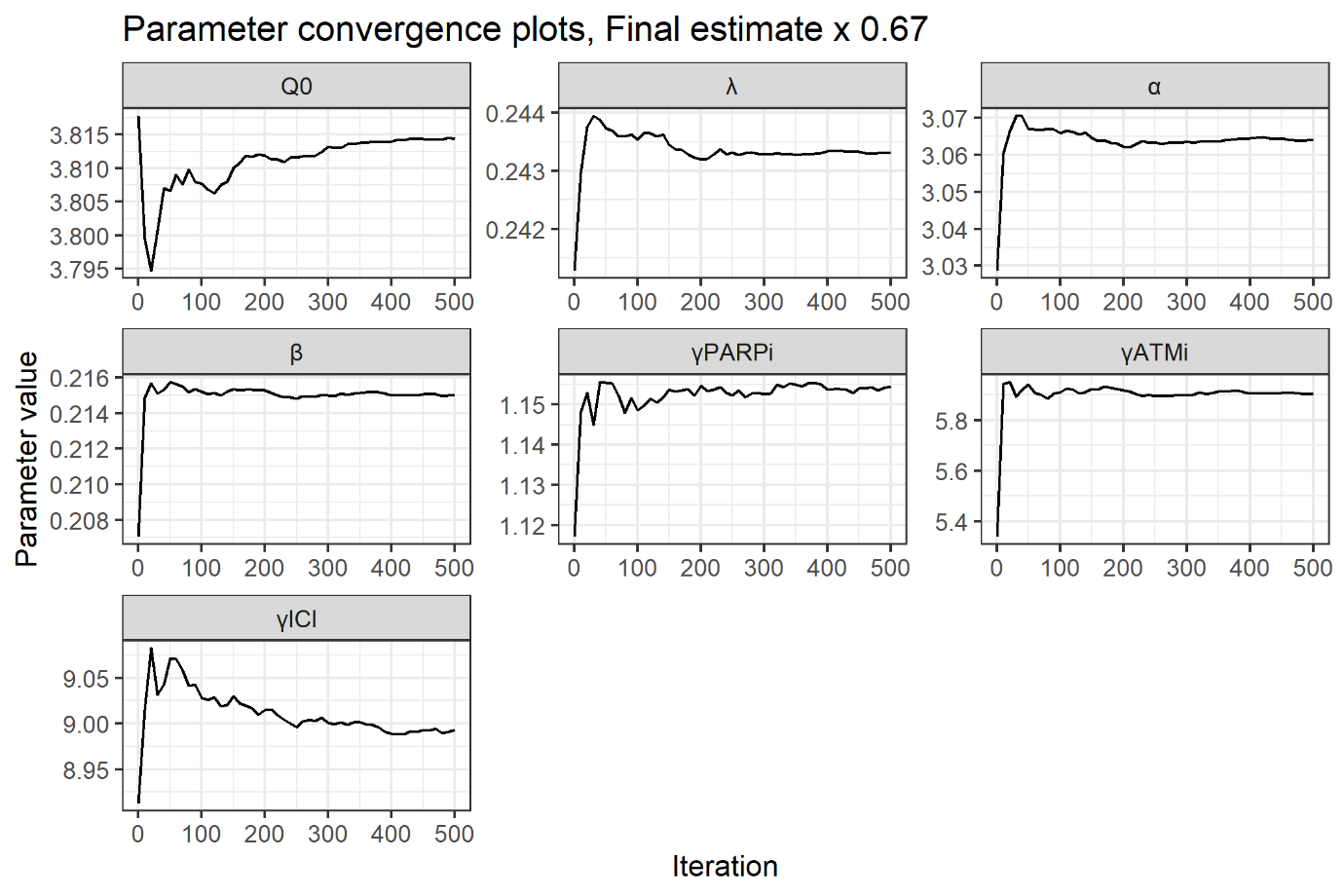
**

**D)**

**
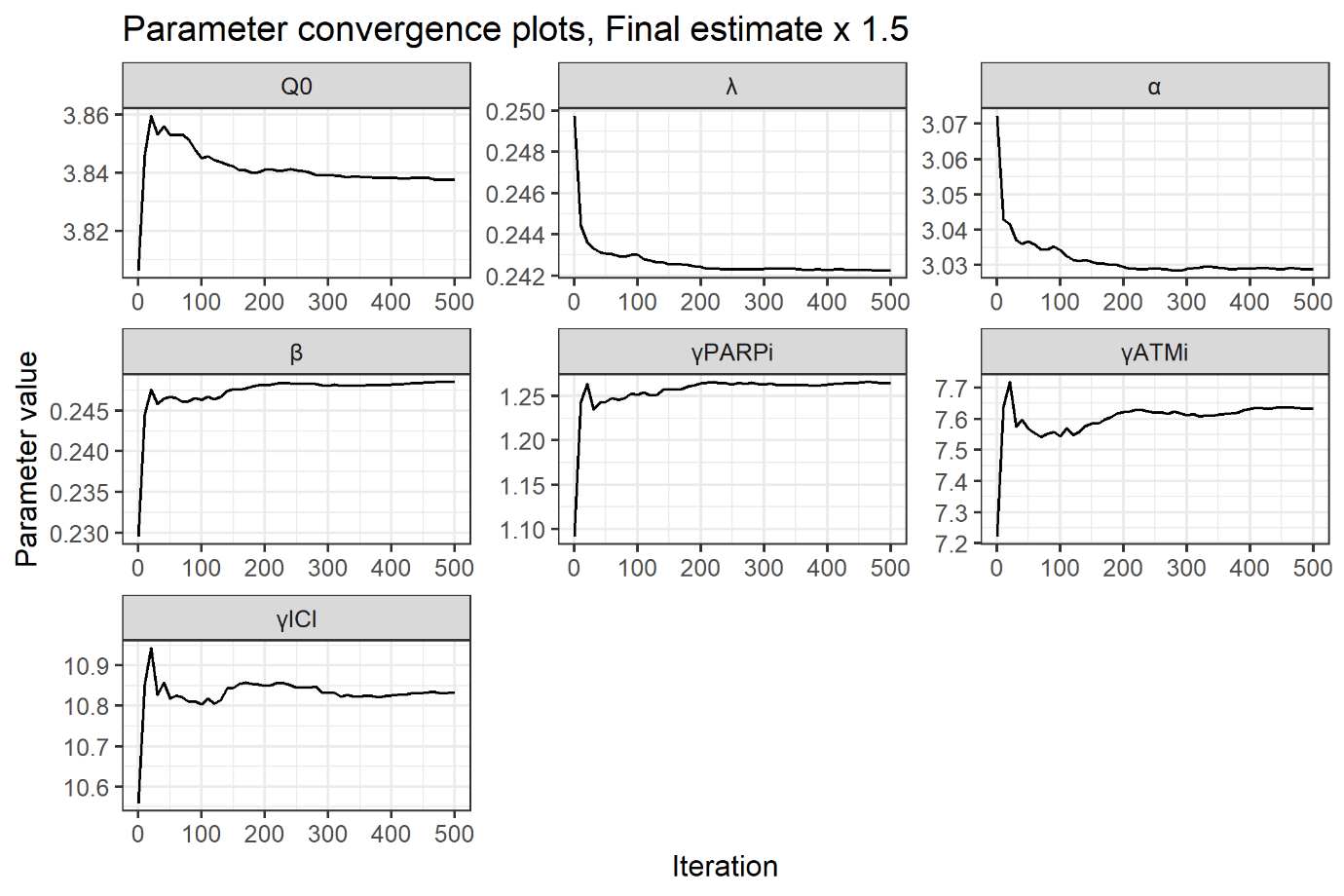
**

**E)**


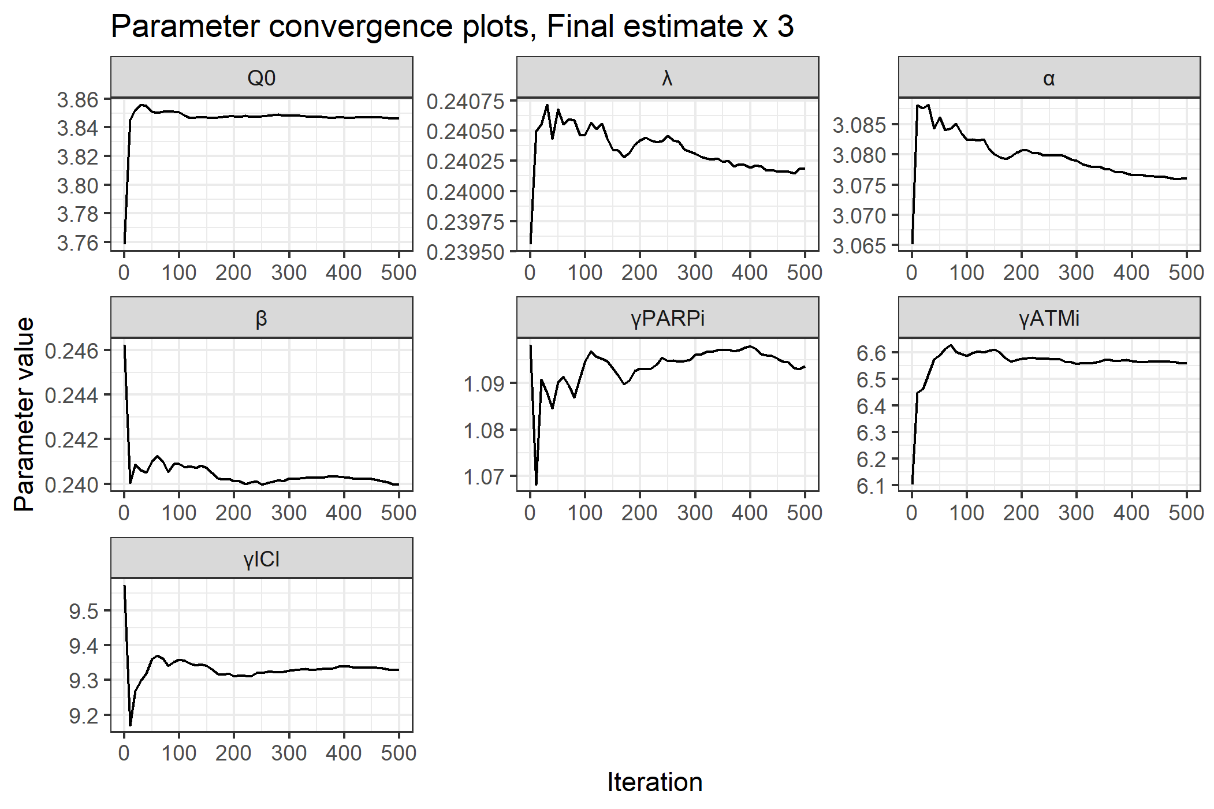


**Figure S3. Additional diagnostic plots. Population predicted tumour diameter vs CWRES (A), Observed vs population predicted diameter (B), Individual predicted tumour diameter vs CWRES (C) and Observed vs individual predicted diameter (D).**

**
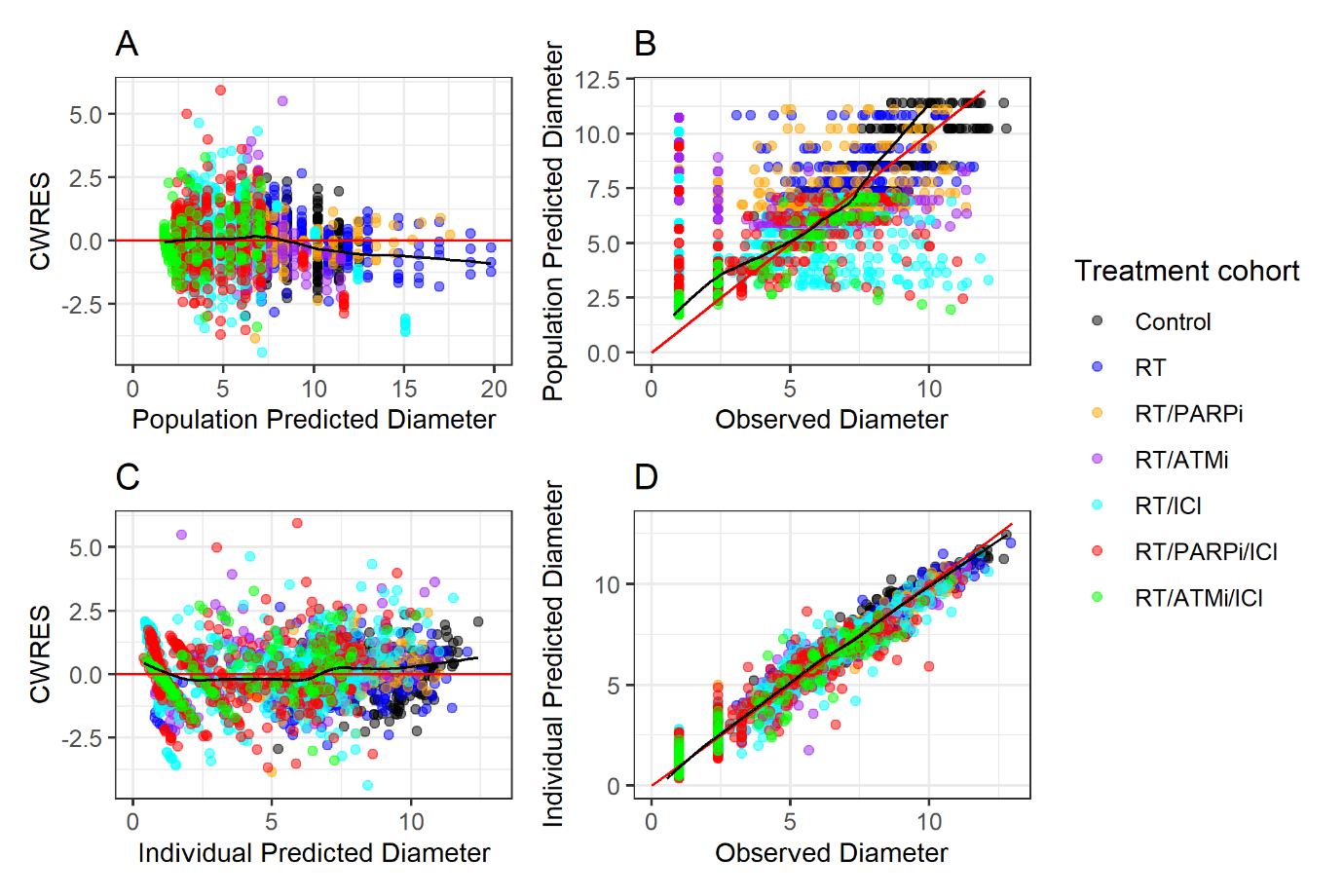
**

**Table S1. Final parameter estimates from additional FOCE-SAEM runs with the initial estimates were perturbed between 1.1 to 1.5 fold.**

| **Parameter** | **Initial estimate** | **Final estimate (SAEM)** | **Initial estimate x 1.1** | **Initial estimate x 1.2** | **Initial estimate x 1.3** | **Initial estimate x 1.4** | **Initial estimate x 1.5** |
| --- | --- | --- | --- | --- | --- | --- | --- |
| Q_0_ | 5 | 3.83 | 3.834 | 3.812 | 3.842 | 3.843 | 3.815 |
| λ | 0.19 | 0.24 | 0.239 | 0.239 | 0.243 | 0.24 | 0.244 |
| α | 3 | 3.06 | 3.084 | 3.042 | 3.038 | 3.038 | 3.053 |
| β | 0.16 | 0.278 | 0.241 | 0.206 | 0.231 | 0.271 | 0.206 |
| γ_PARPi_ | 1 | 2.03 | 1.186 | 1.518 | 1.976 | 1.72 | 1.424 |
| γ_ATMi_ | 1 | 4.34 | 9.003 | 7.85 | 6.861 | 6.137 | 6.714 |
| γ_ICI_ | 1 | 11.9 | 8.918 | 6.56 | 10.777 | 9.675 | 6.752 |
| η_Q0_ | 0.0475 | 0.043 | 0.041 | 0.044 | 0.042 | 0.043 | 0.043 |
| Cor(η_Q0_, η_λ_) | -0.36 | -0.28 | -0.263 | -0.307 | -0.292 | -0.292 | -0.298 |
| η_λ_ | 0.0547 | 0.064 | 0.068 | 0.072 | 0.074 | 0.066 | 0.073 |
| Cor(η_Q0_, η_β_) | 0.03 | -0.04 | 0.019 | -0.005 | -0.025 | 0.005 | -0.02 |
| Cor(η_λ_, η_β_) | -0.03 | -0.11 | -0.238 | -0.205 | -0.283 | -0.193 | -0.24 |
| η_β_ | 2 | 1.25 | 1.286 | 1.219 | 1.12 | 1.315 | 1.222 |
| RV | 0.6 | 0.506 | 0.507 | 0.506 | 0.508 | 0.507 | 0.505 |

**Table S2. Final parameter estimates from an FOCE-SAEM run with the initial estimate set to the final parameter estimates shown in Table 3, comparing with final parameter estimates from FOCE-SAEM runs when perturbing the initial estimate 0.33, 0.67, 1, 1.5 and 3 fold.**

|  |  | **Final estimate** | | | | |
| --- | --- | --- | --- | --- | --- | --- |
| **Parameter** | **Initial estimate** | **x 0.33** | **x 0.67** | **x 1** | **x 1.5** | **x 3** |
| Q_0_ | 3.83 | 3.839 | 3.814 | 3.828 | 3.837 | 3.846 |
| λ | 0.24 | 0.24 | 0.243 | 0.24 | 0.242 | 0.24 |
| α | 3.06 | 3.048 | 3.064 | 3.076 | 3.029 | 3.076 |
| β | 0.278 | 0.237 | 0.215 | 0.24 | 0.249 | 0.24 |
| γ_PARPi_ | 2.03 | 2.338 | 1.155 | 1.426 | 1.265 | 1.094 |
| γ_ATMi_ | 4.34 | 10.154 | 5.903 | 4.873 | 7.632 | 6.558 |
| γ_ICI_ | 11.9 | 8.31 | 8.993 | 9.06 | 10.832 | 9.33 |
| η_Q0_ | 0.043 | 0.043 | 0.043 | 0.042 | 0.042 | 0.042 |
| Cor(η_Q0_, η_λ_) | -0.28 | -0.306 | -0.293 | -0.273 | -0.277 | -0.275 |
| η_λ_ | 0.064 | 0.075 | 0.073 | 0.069 | 0.069 | 0.068 |
| Cor(η_Q0_, η_β_) | -0.04 | 0.008 | -0.016 | -0.041 | 0 | 0.016 |
| Cor(η_λ_, η_β_) | -0.11 | -0.259 | -0.222 | -0.274 | -0.162 | -0.228 |
| η_β_ | 1.25 | 1.218 | 1.163 | 1.272 | 1.184 | 1.289 |
| RV | 0.506 | 0.507 | 0.507 | 0.507 | 0.507 | 3.846 |
